# Synthesis, identification, chiral separation and crystal structure of (3*R*,4*R*,7*S*,8*S*)-3,4,7,8-tetrachlorodecane and its stereoisomers

**DOI:** 10.1016/j.heliyon.2023.e16987

**Published:** 2023-06-03

**Authors:** Solveig Valderhaug, Natalie Paškanová, Jiří Tůma, Jana Herciková, Václav Eigner, Huiling Liu, Alexey Gorovoy, Jon Eigill Johansen, Odd Reidar Gautun

**Affiliations:** a Department of Chemistry, Norwegian University of Science and Technology (NTNU), Høgskoleringen 5, NO-7491, Trondheim, Norway; b Chiron AS, Stiklestadveien 1, NO-7041 Trondheim, Norway; c Department of Organic Chemistry, University of Chemistry and Technology, Technická 5, 16628, Prague, Czech Republic; d Department of Solid State Chemistry, University of Chemistry and Technology, Technická 5, 16628, Prague, Czech Republic

**Keywords:** Chlorinated paraffins, Chiral supercritical fluid chromatography, Amylose, X-ray diffraction, Gas chromatography

## Abstract

Chlorinated paraffins (CPs) are a notoriously known class of compounds that stand amongst the most wide-spread persistent organic pollutants. Therefore, their reliable, repeatable, and reproducible quantitative analysis using well-defined reference standards is of utmost importance. In view of the increasing demand for constitutionally and stereochemically defined CP standards, we have synthesized a stereoisomeric mixture of 3,4,7,8-tetrachlorodecane. One stereoisomer – (3*R*,4*R*,7*S*,8*S*)-3,4,7,8-tetrachlorodecane was separated from the mixture, and enriched fractions of residual stereoisomers were achieved through crystallisation of the residual mother liquors. The molecular structure of the single isolated stereoisomer was confirmed through single-crystal X-ray crystallographic data. One fraction of 3,4,7,8-tetrachlorodecane stereoisomers was successfully separated on a chiral stationary phase using supercritical fluid chromatography hyphenated to mass spectrometry (column: Chiral ART Amylose-C; mobile phase: CO_2_/MeOH (96/4 v/v) with 0.1% diethylamine). The reported separation of stereoisomers is unprecedented in CP analysis so far.

## Highlights

• A chlorinated paraffin stereoisomer with four stereogenic centers was isolated.
• Its crystal structure was confirmed by X-ray crystallography.
• Chiral separation of a diastereomer chlorinated paraffin mixture was achieved.
• Obtained data agrees with the concept of stereospecific chlorination of polyenes.

## Graphical abstract

**Figure:**
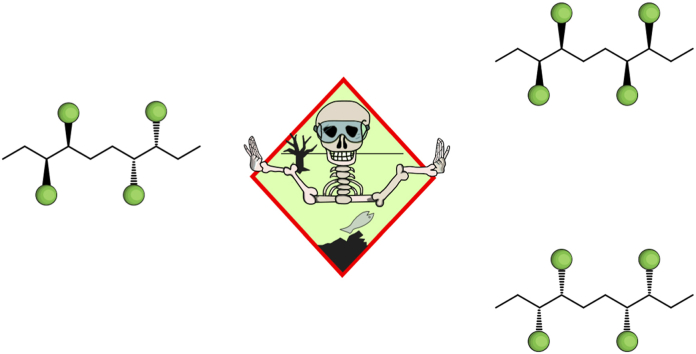

## 1 Introduction

Persistent organic pollutants (POPs) are synthetic chemicals that resist environmental degradation and are toxic to humans and the environment [1]. Through biomagnification, the concentrations in humans and other high trophic species may reach harmful levels if not properly regulated [2]. The Stockholm convention was a global environmental treaty established to regulate or eliminate the use of POPs [3,4]. The listed chemicals include polyhalogenated pesticides and insecticides, polychlorinated biphenyls, perfluorooctanoic acid and short-chain chlorinated paraffins (SCCPs, C_10-13_). SCCPs are a subclass of chlorinated paraffins (CPs), which also consist of medium-chain CPs (MCCPs, C_14-17_) and long-chain CPs (LCCPs, C_≥18_) and very short chain CPs (vSCCPs, C_≤9_) [5].

Monitoring CPs in the environment is troublesome [6], as they are composed of mixtures of potentially several thousands of positional isomers. It is natural that such compounds can bear secondary -CHCl-moieties that constitute chiral centers, thus, making the molecule chiral as a whole (with exception of meso forms). The established methods of analysis of CPs include liquid chromatography (LC) and gas chromatography (GC), with detection techniques solely based on mass spectrometry (MS) [7]. The use of traditional UV–Vis detection is suboptimal as CPs possess only low-absorbent chromophores that exhibit absorption maxima in the UV region that are commonly overlapped with bands of other components (e.g., chromatographic solvents). The current most commonly applied method for analysis of CP mixtures is GC coupled to negative chemical ionization mass spectrometry. This approach is, however, limited to CPs with at least 5 chlorine atoms [8]. Another reported method is based on LC coupled electrospray ionization-Orbitrap mass spectrometry. This approach enables simultaneous detection of SCCPs, MCCPs and LCCPs with 10 to 36 carbons [9]. Nevertheless, despite these progresses, the overall complexity of CP mixtures causes inter-laboratory variance in quantitative measurements. This is especially pronounced if the quantification standard does not match the analyte [10]. Regrettably, the availability of reference standards for CPs is low, particularly for constitutionally defined and enantiomerically pure CPs [11,12]. The scarcity of constitutionally and stereochemically defined CPs reflects the paucity of literature regarding their synthesis and, to the best of our knowledge, only one single CP compound has been reported with X-ray crystallographic data [13,14]. These types of standards are needed to improve quantitative accuracy in the investigation of specific structural impact on persistence, toxicity, degradation and mechanical studies [12,15,16], to help unravel the environmental fate of CPs.

The chiral nature of CPs is a strikingly overlooked phenomenon. Despite the undisputable fact that stereospecific recognition of chiral molecules is an important issue in various aspects of chemistry and life sciences [17,18], not a single chiral chromatographic study of CP mixtures has been reported so far. The contemporary analytical separation science offers a plethora of methods for chiral separation based on chromatographic or electromigration techniques. One of these techniques, supercritical fluid chromatography (SFC), underwent a significant resurgence of interest in recent years thanks to the development of modern instruments to comply with current expectations in terms of robustness, increased sample throughput, and sensitivity [19]. SFC employs pressurized, supercritical carbon dioxide as major component of the mobile phase mixed with a polar modifier (typically alcohol), thus producing minimal amount of toxic waste. For these reasons, SFC is viewed as an eco-friendly chromatographic method that represents the future of separation science. Therefore, finding efficient protocols for both chiral and achiral separation in SFC mode is of utmost importance with respect to both its time and waste management benefits. In spite of these major advantages, it is worth noting that SFC, once hyphenated to MS detection, struggles with ionization efficiency. Hence, thorough optimization of MS detection conditions (e.g., cone voltage settings, composition of make-up solvent, etc.) is commonly required. The overall struggle for efficient MS detection is a major obstacle in broad application of the SFC-MS technique. SFC-MS has already been successfully employed in analysis of several halogenated environmental pollutants [20]. However, implementation of SFC-MS in separation of CPs and, notably, any attempt for chiral separation of CP stereoisomers has not been reported up to date.

Herein, we present a methodological approach to synthesis, separation, and characterization of one new model SCCP compound – 3,4,7,8-tetrachlorodecane (**4**). We found that the crude CP mixture contains 7 different stereoisomers, including one meso form – (3*R*,4*R*,7*S*,8*S*)-3,4,7,8-tetrachlorodecane (**4a**). The title compound was purified by recrystallization and characterized using X-ray crystallography. Furthermore, one of the isolated fractions of stereoisomers was successfully separated on a chiral stationary phase using SFC-MS. The data are consistent with parallel GC-MS and NMR (nuclear magnetic resonance) analyses. We believe that the presented SFC approach is the very first protocol for chiral separation of CP stereoisomers reported so far.

## 2 Results and discussion

### 2.1 Synthesis

**Scheme 1:**
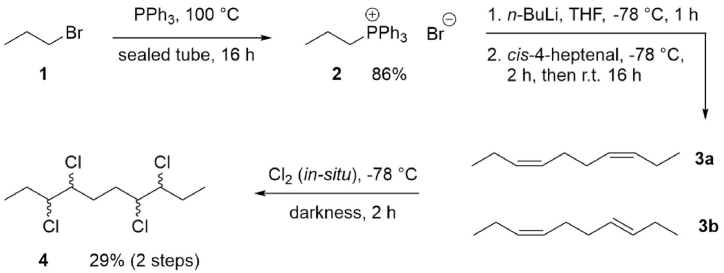
Synthesis of **4**, obtained as an isomer mixture.

The synthesis of the isomer mixture of 3,4,7,8-tetrachlorodecane (**4**) was carried out over three steps, including preparation of phosphonium salt **2** [21], Wittig olefination [22] and subsequent chlorination (Scheme 1).

Through a Wittig olefination with the non-stabilised ylide resulting from **2** and *cis*-4-heptenal, the product **3** was expected to show a high *Z*/*E* ratio [23]. Due to extended overlap (unresolved multiplets) between the *Z* and the *E*-isomer in the ^1^H NMR spectrum of **3**, the *Z*/*E* selectivity could not be accurately determined but suggested the presence of both. Previous work on similar compounds have demonstrated a high degree of *Z*-selectivity [[24], [25], [26]].

**Scheme 2:**
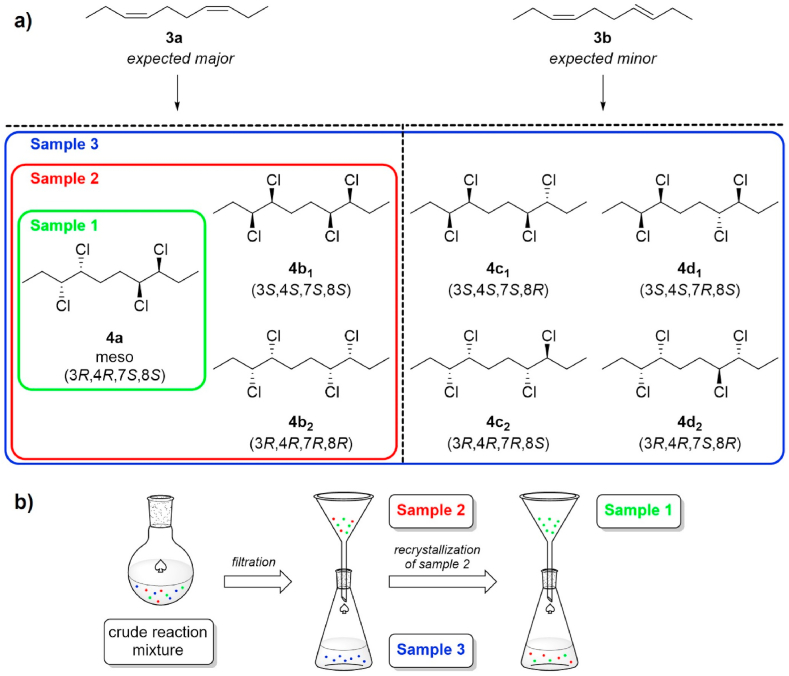
(a) Stereoisomer products of 3,4,7,8-tetrachlorodecane (**4**) from the different expected isomer products of 3,7-decadiene, assuming the chlorination follows anti-addition only. Diene **3a** is expected as the major component according to the reaction mechanism [23]. (b) Schematic description of the isolation of various CP samples from the crude reaction mixture.

The chlorination of double bonds is known to follow *anti*-addition [27], which gives rise to a product mixture (**4a-d**) as shown in Scheme 2a, given only *anti*-addition of chlorine occurs. In the case of dienes **3a-b**, the chlorination was carried out using our pre-optimized protocol (see Materials and methods section) which utilizes *in-situ* generated chlorine gas as chlorination agent [28]. The reaction yielded a crude white precipitate and a residual filtrate – both containing mixtures of the target CP (Scheme 2b).

### 2.2 Sample treatment and GC-MS analysis

The solid that precipitated directly from the reaction mixture was collected as sample 2 (see Scheme 2b) and provided a diastereomeric mixture of **4** in a 21% yield over two steps (Wittig reaction + chlorination). The GC-MS chromatogram (see Table 1 and SI, Fig. S15) showed the presence of two major peaks at *t*_R_ = 15.9 and 16.1 min in a 36:64 ratio, both with *m*/*z* = 242.1, which corresponds to [M − HCl]^+·^ ion. After recrystallization of sample 2 from isopropyl alcohol, the resulting crystals (sample 1; see Scheme 2b) showed only one peak in GC-MS (see SI, Fig. S10) at *t*_R_ = 16.3 min (6% yield over two steps). Further recrystallizations of the mother liquor from sample 2 failed to afford further portions of the purified product (sample 1).

The filtrate of the original reaction mixture (sample 3; see Scheme 2b) was concentrated and purified by dry column vacuum chromatography [17]. The GC-MS (SI, Fig. S20) revealed four major peaks at *t*_R_ = 15.6, 15.7, 16.0 and 16.2 min in an 8:20:42:27 ratio all with *m*/*z* = 242.1, in addition to ∼3% of overchlorinated products *t*_R_ = 18.4–19.0 min (*m*/*z* = 276.0). Since sample 3 exhibited four distinctive signals in GC-MS, it can be concluded that it consisted of all four possible diastereomers **4a-d**, as depicted in Scheme 2a. As discussed in the previous paragraph, the two major peaks from sample 3 were also found in the sample 2, *i.e.*, the solids isolated directly from the reaction mixture. Since sample 3 featured all four diastereomers, it indicated that the sample 2 held exactly two diastereomers, and, by extension, that sample 1 contained one diastereomer only (see Table 1). The relative ratio of the peaks in sample 3 in conjunction with the two peaks found in sample 2 corresponds to the expected abundance of the diene precursor **3a** over **3b**, which resulted in predominant formation of **4a** and **4b** stereoisomers, respectively. Therefore, it can be concluded that the stereoisomers **4a** and **4b** were indeed the prevalent stereoisomers found in the CP reaction mixture.

**Table 1:** Summary of GC-MS analysis of samples 1–3.

Sample	retention time [min] *relative intensity*			
1	–	–	–	16.3 *100*
2	–	–	15.9 *36*	16.1 *64*
3^a^	15.5 *8*	15.7 *20*	16.0 *42*	16.2 *27*

a Sample 3 contained ∼3% of overchlorinated products.

### 2.3 Characterization of samples 1-3

In order to determine the composition of samples 1–3 and to conclusively prove the structure of the isolated stereoisomers, X-ray diffraction analysis and separation using supercritical fluid chromatography with a chiral column were performed.

#### 2.3.1 X-ray diffraction data of **4a**

**Fig. 1:**
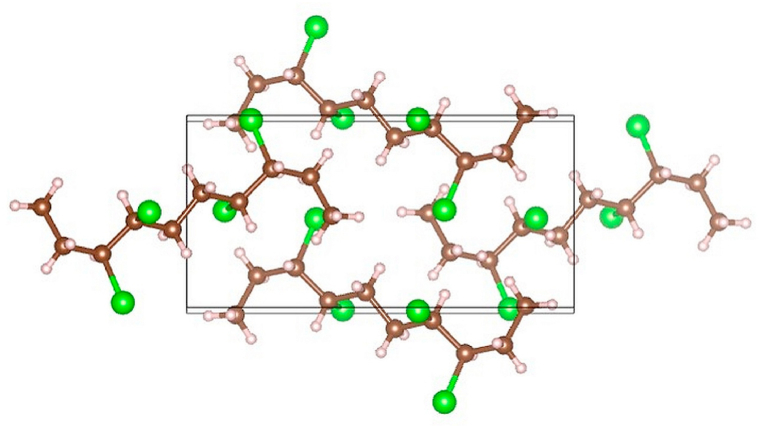
Graphical depiction of **4a** (sample 1) unit cell from X-ray diffraction.

The crystals collected from recrystallization as sample 1 were of excellent quality for single-crystal X-ray diffraction. This way, the molecular structure of the single compound present in sample 1 (see Table 1 and Fig. 1) was assigned as **4a**. This finding is in agreement with the expected structure of the reaction products outlined in Scheme 2a.

#### 2.3.2 Chiral separation of 3,4,7,8-tetrachlorodecane isomers

The aim of the chiral separation was to exploit a complementary method to the performed GC-MS analysis for proving the composition of the samples 1–3, and that would yield information about the enantiomeric composition as well. The chiral separation was performed on Acquity UltraPerformance Convergence Chromatography TM (UPC^2^) from Waters (Milford, MA, USA) by means of SFC with MS (ESI-) detection using Chiral ART Amylose-C (250 × 4.6 mm, S-5 μm) from YMC Europe GmbH (Dinslaken, Germany) column as a stationary phase. Detailed instrumentation is described in the Materials and methods section.

##### 2.3.2.1 Optimization of MS detection

The MS detection of CPs is generally troublesome due to their poor ionization and the presence of multiple isotopic peaks. Therefore, prior to the chiral separation itself, optimization of the MS detection and influence of the make-up solvent was determined using pure compound **4a** (sample 1). The initial setting of capillary voltage (from 1.5 to 0.4 kV) and cone voltage (from 30 to 5 V) for negative ionization mode were set. The most abundant ionic adducts detected in the MS spectrum based on the nature of the make-up solvent are shown in Table 2.

**Table 2:** Influence of the make-up solvent on the MS detection in SFC-MS.

Entry	make-up solvent composition	additional ion *m*/*z* (theoretical)	adduct formed *m/z* (theoretical)	ion found
1	MeOH:H_2_O (9:1, v:v) +0.1% HCOOH	HCOO^−^ 45.0	[M + HCOO^−^]^-^ 325.0	324.9
2	MeOH:H_2_O (9:1, v:v) +0.1% CH_3_COOH	CH_3_COO^−^ 59.0	[M + CH_3_COO^−^]^-^ 339.0	339.0
3	MeOH:H_2_O (9:1, v:v) +0.1% NH_4_OH	CH_3_OCOO^−^ 75.0	[M + CH_3_OCOO^−^]^-^355.0	355.0

**Scheme 3:**
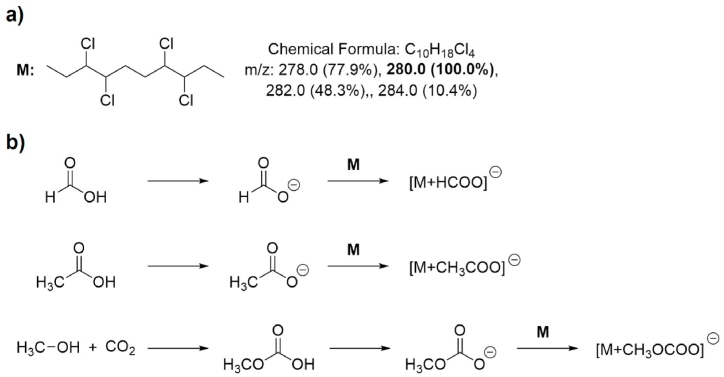
(a) Structure of the molecular ion(s) of neat 3,4,7,8-tetrachlorodecane. (b) Proposed mechanism of adducts found in MS.

The adducts found in the MS spectra stem from addition of the CP (most abundant mass = 280.0) and corresponding conjugate base to the acid in the make-up solvent. Upon using NH_4_OH additive (Table 2, Entry 3) instead of formic (Table 2, Entry 1) or acetic acid (Table 2, Entry 2), an adduct resulting from *in-situ* reaction between methanol and CO_2_ from the mobile phase was formed (Scheme 3).

##### 2.3.2.2 Development of enantioseparation method

**Fig. 2:**
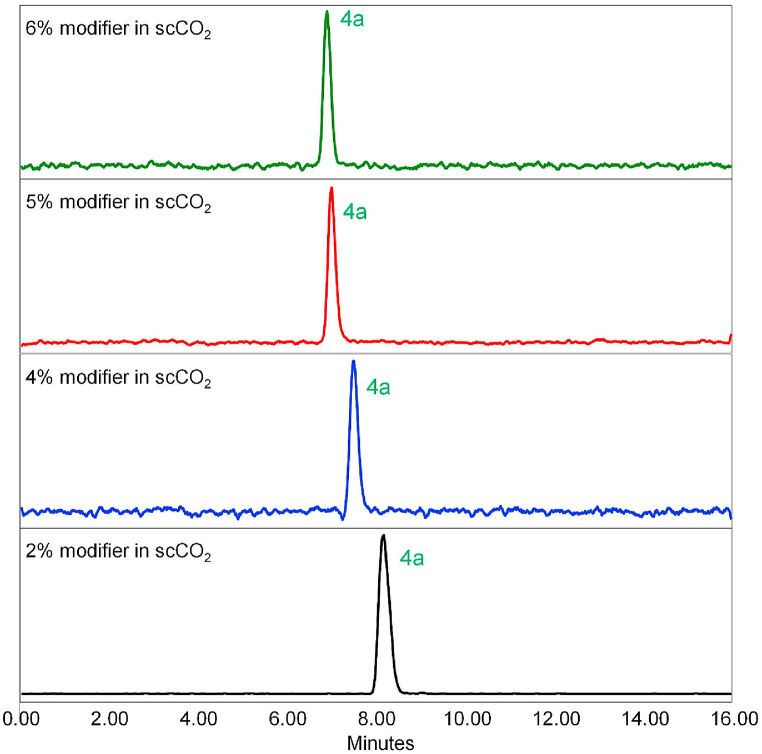
SFC-MS chromatograms of sample 1 measured with different ratio of CO_2_ and modifier (MeOH) with 0.1% DEA additive.

**Fig. 3:**
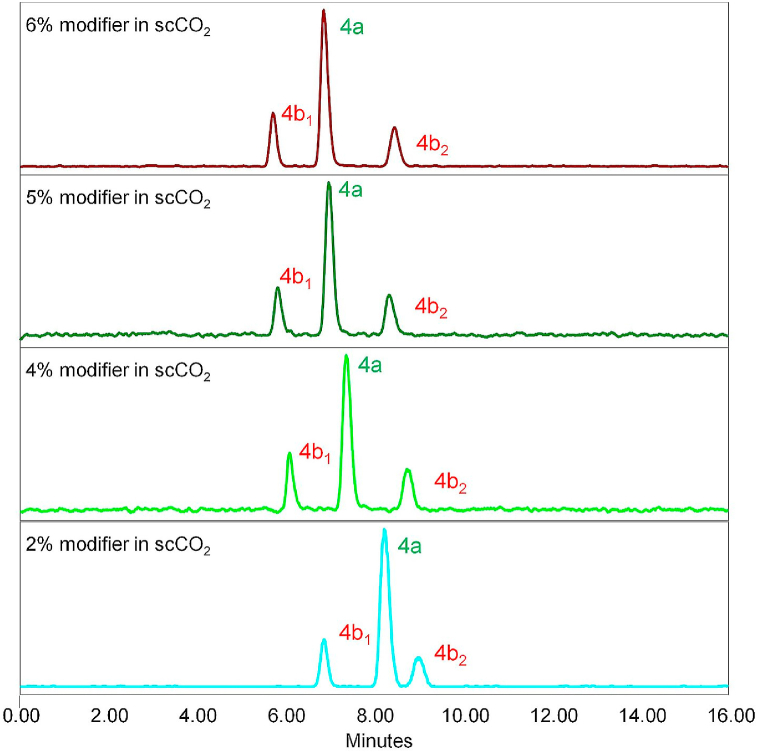
SFC-MS chromatograms of sample 2 measured with different ratio of CO_2_ and modifier (MeOH) with 0.1% DEA additive.

**Fig. 4:**
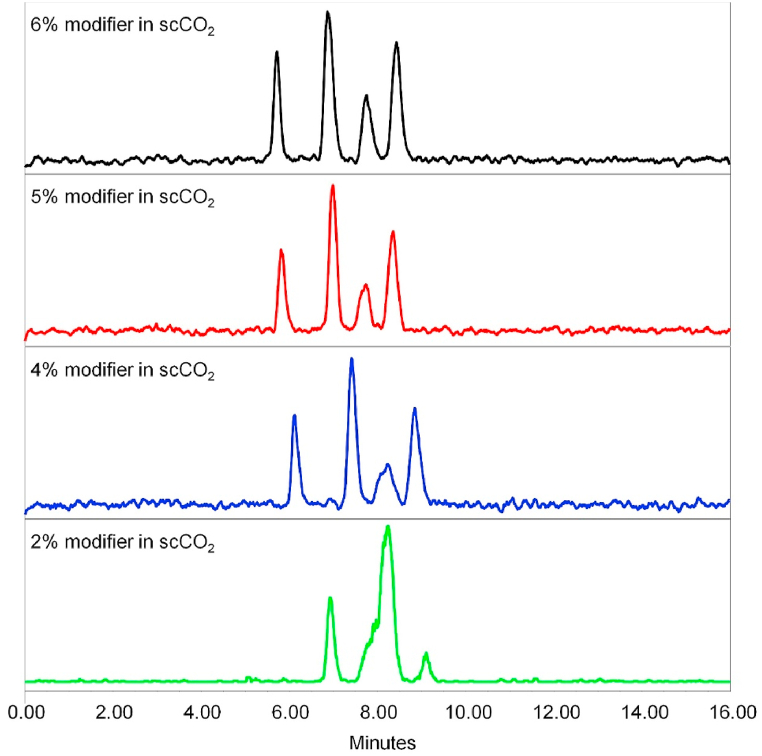
SFC-MS chromatograms of sample 3 measured with different ratio of CO_2_ and modifier (MeOH) with 0.1% DEA additive. Peaks are not assigned to the specific stereoisomers due to insufficient separation.

**Fig. 5:**
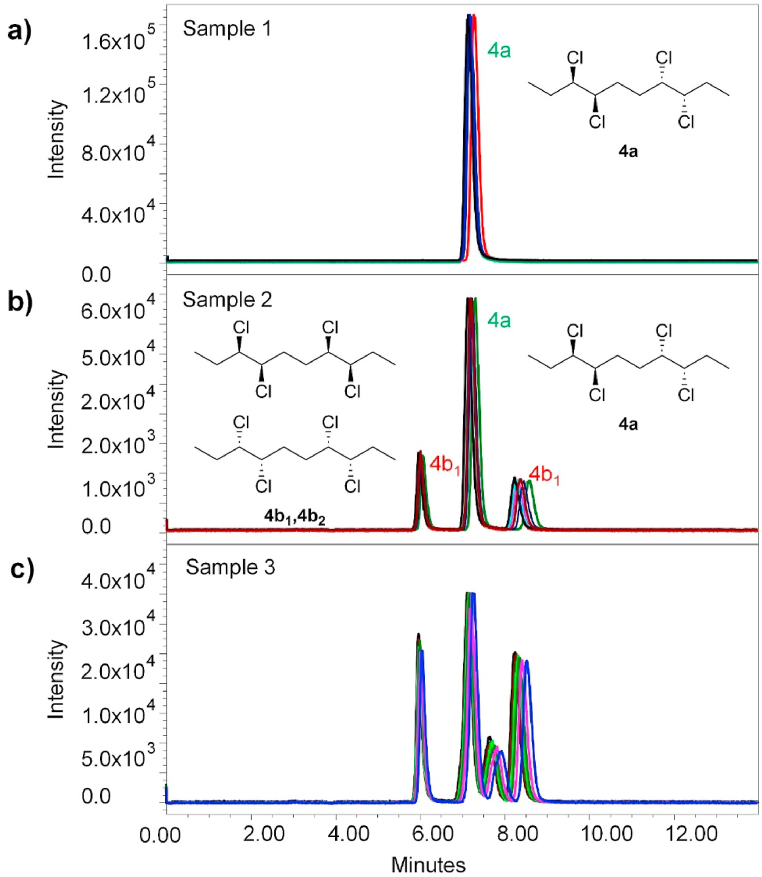
SFC-MS chromatograms of (a) sample 1; (b) sample 2; (c) sample 3. The enantiomers **4b**_**1**_ and **4b**_**2**_ are not specifically assigned to their chromatographic peaks. The indexing refers to the order they were eluted from the chromatographic column. Peaks for sample 3 are not assigned to the specific stereoisomers due to insufficient separation.

In preliminary tests, various polar organic modifiers (propan-2-ol, ethanol, methanol and acetonitrile) were introduced into the bulk supercritical carbon dioxide mobile phase. To enhance enantioseparation and suppress possible non-enantioselective interactions of the analyte with the chiral stationary phase (e.g., residual silanol groups on the silica surface), basic (isopropylamine, diethylamine) and acidic (formic acid) additives, to the mobile phase, were screened. The best results were found using methanol as modifier with 0.1% diethylamine (DEA) additive. Therefore, optimization of the modifier concentration (MeOH) for the separation of samples 1–3 was carried out as shown in Fig. 2, Fig. 3, Fig. 4. Based on the obtained chromatograms, the use of CO_2_/MeOH = 96:4 + 0.1% DEA was assessed as the optimized mobile phase for the given separation as this mobile phase showed a hint of separation of the third peak eluted in the case of sample 3 (Fig. 4). The repeatability of the retention time as well as the resolution factor for all samples was checked out in five consecutive runs (Fig. 5a–c).

##### 2.3.2.3 Chiral separation using optimized conditions

**Fig. 6:**
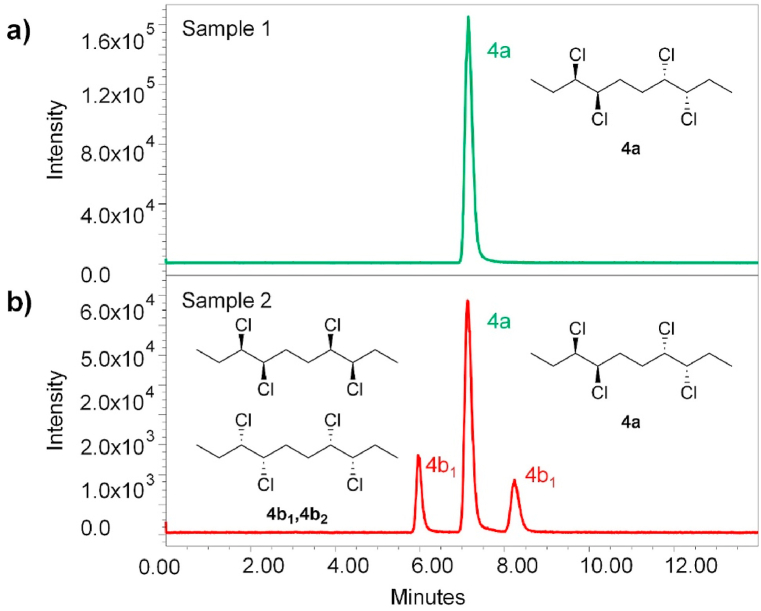
SFC-MS chromatograms of (a) sample 1; (b) sample 2. The enantiomers **4b**_**1**_ and **4b**_**2**_ are not specifically assigned to their chromatographic peaks. The indexing refers to the order that they were eluted from the chromatographic column.

The SFC separation of sample 1 yielded a single peak at *t*_**4a**_ = 7.2 min (see Figs. 2 and 6a and Table 3). This result, in conjunction with the X-ray crystallography data, further proves that the sample 1 holds a single stereoisomer (meso form **4a**) and is of very high purity overall. Afterwards, sample 2 was tested using the same separation conditions. Three individual peaks with relative intensity 17:66:17 and retention times *t*_**4b1**_ = 6.0 min, *t*_**4a**_ = 7.2 min, and *t*_**4b2**_ = 8.4 min, respectively were obtained (Fig. 6b). This correlates with the expected presence of three stereoisomers (**4a**, **4b**_**1**_, **4b**_**2**_) in the sample 2. All the stereoisomers were baseline separated. Furthermore, their relative ratio was in good agreement with the GC-MS analysis, which determined the ratio of **4a**:**4b** in sample 2 as 64:36 (hence **4a**:**4b**_**1**_:**4b**_**2**_ = 64:18:18). To the best of our knowledge, this is the very first successful chiral separation of CP stereoisomers reported so far. Not only diastereomeric separation of **4a** and **4b** was achieved, but also a very good enantioseparation of individual **4b**_**1**_ and **4b**_**2**_ enantiomers with excellent selectivity *α* = 1.45 and resolution *R*_S_ = 7.00 was reached.

**Table 3:** SFC-MS separation of samples 1 and 2.

sample	compound	*t*_R_ [min]	rel. abundance
1	**4a**	7.19	100
2^a^	**4b**_**1**_	6.02	16.9
	**4a**	7.20	66.6
	**4b**_**2**_	8.37	16.6

a GC-MS of sample 2 assessed **4a**:**4b** = 64:36, hence **4a**:**4b**_**1**_:**4b**_**2**_ = 64:18:18.

The SFC separation of sample 3, which contains a total sum of 7 diastereomers (**4a**, **4b**_**1**_, **4b**_**2**_, **4c**_**1**_, **4c**_**2**_, **4d**_**1**_, **4d**_**2**_), yielded four individual peaks, thus clearly showing co-elution of several stereoisomers at the same time. Therefore, the identification of individual compounds and determination of their relative abundance was not possible with the applied conditions. However, based on the good agreement of SFC results with GC-MS data for sample 2, it can be assumed that the relative ratio of the stereoisomers in the sample 3 correlates with the GC-MS data as well (see Table 1). The chromatograms for sample 3 are shown in Fig. 4, Fig. 5.

#### 2.3.3 NMR spectroscopy

**Fig. 7:**
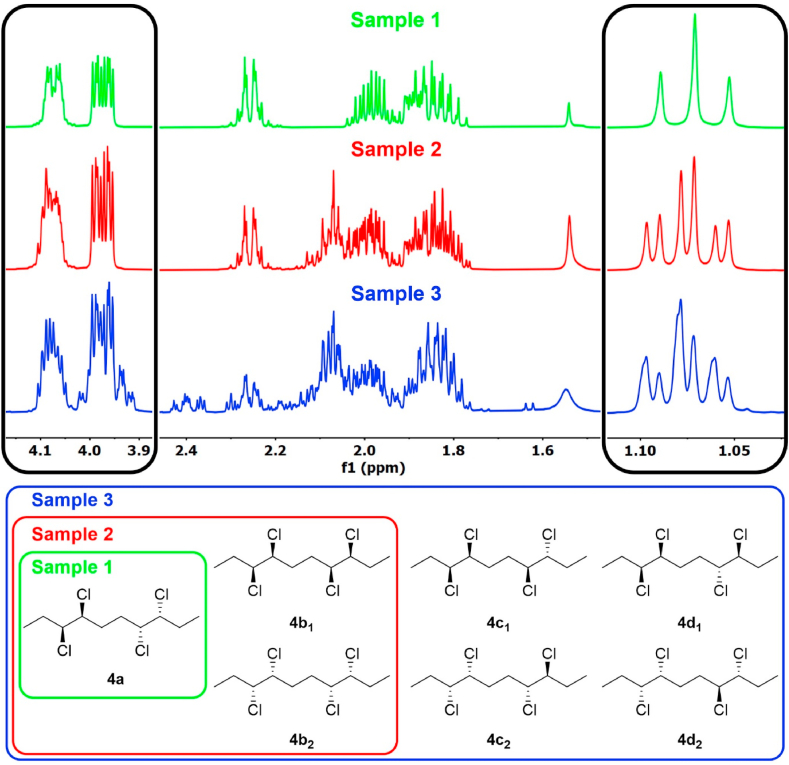
^1^H NMR spectra (400 MHz, CDCl_3_) of samples 1–3, containing different number of isomers of **4**. The regions of interest (1.04–1.12 ppm and 3.90–4.15 ppm) are zoomed in.

The samples 1–3 were analyzed by ^1^H NMR spectroscopy (Fig. 7). One distinct methyl group was found at *δ* 1.07 ppm for sample 1, whereas two methyl groups, *δ* 1.08 ppm and *δ* 1.07 ppm were apparent in the spectrum of sample 2. Poor resolution of peaks in sample 3 indicates the presence of additional isomers. Similar signal distortion pattern was found in the region of protons adjacent to chlorine atoms (*δ* 3.9–4.1 ppm). With increasing complexity of the CP mixture, the signals in this area are less symmetric. These results are in agreement with the other analyses performed, *i.e.*, GC-MS, SFC-MS, and X-ray crystallography.

**Fig. 8:**
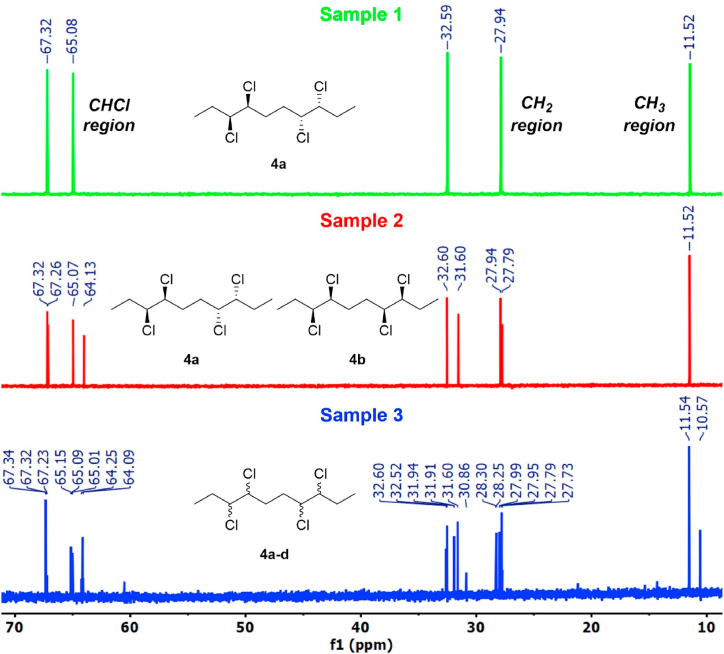
^13^C NMR of samples 1–3 (400 MHz, CDCl_3_).

The samples 1–3 were further analyzed using ^13^C NMR spectroscopy. The spectrum of sample 2 unveiled the presence of two diastereomers, one which was **4a** based on the spectrum of sample 1 as a reference. The spectrum of sample 3 exhibited multiple signals, including the ones ascribed to the **4a** and **4b** stereoisomers. The growing complexity of the spectra is in agreement with the postulated composition of the respective samples 1–3, thus further supporting the conclusions drawn from the GC-MS and SFC-MS analyses. The notable increase of individual, non-overlapping signals in the sample 3 is ascribed to the fact that isomers **4c** and **4d** are not symmetric, thus theoretically having ten individual carbon signals each. On the other hand, compound **4a** has a center of symmetry and **4b** is *C*_2_ symmetric. Therefore, both **4a** and **4b** exhibit only five carbon signals each, of which eight are distinguished in the spectrum of sample 2 while the respective methyl signals are merged at 11.5 ppm. The spectra are shown in Fig. 8.

## 3 Conclusions

A novel diastereomeric mixture of CP **4** was synthesized for the purpose of being used as a reference standard for environmental analysis. The meso compound **4a** was isolated and its structure confirmed by X-ray crystallography. Chiral separation of components **4a**, **4b**_**1**_ and **4b**_**2**_, using supercritical fluid chromatography, was achieved with baseline separation of the individual peaks. The prevalence of mainly **4a** and **4b** in the reaction mixture shows *ex post facto* evidence of **3a** as the major precursor afforded by Wittig olefination prior to the *anti*-addition of chlorine to yield the target CP products.

## 4 Materials and methods

### 4.1 Materials and physical measurements

Propyl bromide, *n*-butyllithium (2.5 M in hexanes) and *cis-*4-heptenal were purchased from Merck (Darmstadt, Germany). Triphenylphosphine and trichloroisocyanuric acid were purchased from Fluorochem (Hadfield, UK). Dry column vacuum chromatography (DCVC) [29] was carried out with silica gel 60 (0.015–0.040 mm, Merck, Darmstadt, Germany).

#### 4.1.1 Instrumentation for chiral separation

Acquity UltraPerformance Convergence Chromatography TM (UPC^2^) from Waters (Milford, MA, USA) was used for the chiral separation. The system featured a binary solvent delivery pump, an autosampler, an automated back-pressure regulator, a column oven compatible with 250 mm long columns, and a photodiode array detector. An isocratic solvent manager was inserted to deliver the make-up solvent to the column effluent prior to mass detection. The mass detector used was a single quadrupole (QDa, Waters, Milford, MA, USA) with electrospray ionization. A mixture of methanol and water (9/1, v/v) with 0.1% of formic acid as the make-up solvent, with 0.4 mL/min flow rate, was used to compensate for poor ionization of the analytes in the respective (non-polar) mobile phase. The complete setting of parameters of ionization was as follows: probe temperature 600 °C, source temperature 120 °C, nitrogen flow rate 5 L/min, capillary voltage 0.8 kV, cone voltage 7 V. The analytes were detected in negative ionization mode by single ion monitoring (SIM) as adducts of analyte and components from the mobile phase and the make-up solvent (see Table 2). Adjustment of the composition of the make-up solvent and parameters of ionization and mass detection are discussed in the Electronic Supplementary Material. The chromatographic system was controlled by Empower® 3 software (Waters, Milford, MA, USA).

The column Chiral ART Amylose-C (250 × 4.6 mm, S-5 μm) from YMC Europe GmbH (Dinslaken, Germany) was used as stationary phase. CO_2_/MeOH (96/4 v/v) with 0.1% DEA was used as the optimal composition of the mobile phase. The chromatographic measurements were performed at a flow rate of 1 mL/min, the column temperature was 35 °C, back pressure was 2000 psi (∼138 bar). The stock solutions of analytes were prepared in MeOH at concentrations of 1–2 mg/mL and stored at 5 °C. Solutions of analytes at a concentration of 500 μg/mL were used for the individual analyses. Void volume was determined as first negative peak of blank sample (solvent injection). The injection volume was 2 μL and the autosampler temperature was set to 10 °C. All measurements were performed at least five times to test reproducibility of the performed analyses.

#### 4.1.2 XRD instrumentation

A single crystal of **4a** measuring 0.41 × 0.29 × 0.26 mm was mounted on a loop and measured on Bruker D8 Venture, using Cu-Kα radiation from sealed micro-focused X-ray tube (*λ* = 1.54178 Å) and Photon CMOS detector. The sample was cooled by an open flow of dry air at 180 K. The crystal belonged to monoclinic system and *P*2_1_/*n* space group. The final unit cell parameters were *a* = 6.3048 (2) Å, *b* = 12.6797 (4) Å, *c* = 8.0997 (3) Å, *β* = 91.4580 (13)°, *Z* = 2, *V* = 647.30 (4) Å^3^. The calculated density was *D*_*c*_ = 1.228 g cm^−3^ and absorption coefficient was *μ*(Cu-Kα) = 0.73 mm^−1^. The data reduction, scaling, and absorption correction were performed using Apex4 [30]. The structure was solved by charge flipping using Superflip [31]. The model was refined on full matrix least squares on *F*^2^ in Crystals [32] using 1225 independent reflections and 64 parameters to final values of *R*_obs_ = 0.028 and *wR*_all_ = 0.077. All hydrogen atoms were visible in difference electron density maps, but according to common practice, they were kept in calculated positions refined with soft restraints and then refined using riding constraints, with *U*_iso_(H) in range of 1.2–1.5 *U*_eq_(C). The structure was deposited into Cambridge Structural Database under number CCDC 2226553.

The melting point was measured with a Gallenkamp melting point apparatus. NMR spectroscopy was performed with a Bruker 400 MHz Avance III HD, Bruker 600 MHz Avance III HD, or JEOL JNM-ECZL400G (400 MHz). Spectral processing was carried out with MestReNova v14.2.1–27684, chemical shifts of NMR signals are listed in ppm using TMS (*δ* = 0.00) as a reference. IR spectroscopy was conducted with a Bruker Alpha FT-IR spectrometer with an ATR-module and were processed in OPUS v.25.

Chromatographic purity was obtained by Agilent 6890 N gas chromatograph equipped with Agilent 7683B injector, Agilent DB-5 fused silica WCOT column (30 m × 0.25 mm × 0.25 μm) and a quadrupole mass spectrometry detector (MSD) Agilent 5975B using electron impact (EI) ionization. The carrier gas used was helium with a flow rate of 1 mL/min. Acquisition was set to full-scan mode. Temperature program: initial 50 °C rising to 175 °C at a rate of 30 °C/min with a hold time of 14 min, followed by a second ramp to 300 °C with a rate of 20 °C/min and a hold time of 5 min.

### 4.2 Synthesis of propyltriphenylphosphonium bromide (2)

A mixture of triphenylphosphine (21.3 g, 81 mmol, 1 eq.) and 1-bromopropane (**1**, 7.4 mL, 81 mmol) was warmed to 100 °C in a pressure vial and stirred for 16 h. The solids were dissolved in MeOH, concentrated under reduced pressure and added on top of a short silica plug. After rinsing with *n*-pentane (100 mL) and elution with 10% MeOH/EtOAc (150 mL), the resulting solution was concentrated under reduced pressure and dried at 60 °C in high vacuum. This afforded propyltriphenylphosphonium bromide (**2**, 27.0 g, 70 mmol, 86%) as a white solid. M.p. 232.9–234.0 °C (toluene, lit [20]. 236.6 °C). ^1^H NMR (400 MHz, DMSO‑*d*_6_) *δ* 7.97–7.90 (m, 3H), 7.89–7.78 (m, 12H), 3.69–3.57 (m, 2H), 1.60 (app. hept, *J* = 7.2 Hz, 2H), 1.11 (app. t, *J* = 7.2 Hz, 3H). The spectrum was in accordance with reported data [33].

### 4.3 Synthesis of deca-3,7-diene (3)

A suspension of **2** (7.06 g, 18 mmol, 1.0 eq.) in anhydrous THF (150 mL) was cooled to −78 °C before an *n*-BuLi solution (2.5 M in hexanes, 8.0 mL, 20 mmol, ∼1.1 eq.) was added dropwise. The mixture was stirred for 1 h, keeping the temperature between −20 °C and −40 °C. The reaction was re-cooled to −78 °C and *cis*-4-heptenal (2.4 mL, 18 mmol, 1.0 eq.) was added dropwise. After stirring for 2 h at −78 °C the reaction mixture was slowly warmed to r.t. and stirred for an additional 16 h. Water (100 mL) and n-pentane (150 mL) were added, and the two layers were separated. The organic layer was washed with water (3 × 100 mL) and brine (100 mL), dried over MgSO_4_, filtered, and concentrated. After adding *n*-pentane (∼30 mL) to the concentrated product solution, the precipitate (triphenylphosphine oxide) was filtered off and the filtrate concentrated under reduced pressure. This was repeated twice. The crude solution was purified by flash column chromatography (FCC, SiO_2_, *n*-pentane, R_f_ ∼0.85) and concentrated under reduced pressure to afford deca-3,7-diene (**3a** and **3b**, ∼1.26 g, 9.1 mmol, 51% estimated from NMR) as a solution in *n*-pentane (total 4.52 g), which was used in the next step without further purification. ^1^H NMR (400 MHz, CDCl_3_) *δ* 5.53–5.28 (m, 4H), 2.12–1.97 (m, 8H), 0.97 (app. t, *J* = 7.5 Hz, 6H). The spectrum was similar to reported data with CCl_4_ as solvent [34].

### 4.4 Synthesis of 3,4,7,8-tetrachlorodecane (4)

The solution of **3** (∼1.26 g, 9.1 mmol) was diluted with *n*-hexane (100 mL) and cooled to −78 °C. In a different flask, aq. HCl (2 M, 10–15 mL) was added dropwise to trichloroisocyanuric acid (∼2 g, 8.6 mmol), creating chlorine gas [28], which was bubbled into the olefin solution in darkness. The chlorine gas supply was removed when the solution turned yellow. After stirring at −78 °C for 2 h in darkness, the reaction mixture was quenched with 1-pentene to consume the excess of chlorine. The formed precipitate (1.07 g, 3.8 mmol) was collected by filtration (sample 2) and the filtrate was concentrated under reduced pressure. The resulting solid was collected for further purification.

Precipitate: The precipitate was recrystallized from *i*-PrOH affording (3*R*,4*R*,7*S*,8*S*) 3,4,7,8-tetrachlorodecane (**4a**, 290 mg, 1.0 mmol, 6% yield over two steps) as a white crystalline solid (sample 1). The mother liquor was partially concentrated, causing further precipitation of white solid to a ∼33:67 (GC-MS) mixture of **4a** and (3R*,4R*,7R*,8R*)3,4,7,8-tetrachlorodecane **4b** (240 mg, 0.9 mmol, 5% yield over two steps). After concentrating the mother liquor, a ∼43:57 (GC-MS) mixture of **4a** and **4b** (540 mg, 1.9 mmol, 11% yield over two steps) was obtained as a white solid. Purification by dry column vacuum chromatography (SiO_2_, *d* = 2 cm, *l* = 5 cm, stepwise elution: *n*-hexane (15 × 5 mL), 5% DCM in *n*-hexane (15 × 5 mL) and 10% DCM in *n*-hexane (15 × 5 mL)) was tested for both mixtures but made insignificant difference to the mixtures’ composition.

Solids obtained from the original filtrate: The residual solid was purified by dry column vacuum chromatography (SiO_2_, *d* = 2 cm, *l* = 5 cm, stepwise elution: *n*-hexane (15 × 5 mL), 5% DCM in *n*-hexane (15 × 5 mL) and 10% DCM in *n*-hexane (15 × 5 mL)). And afforded 8:20:42:27 (GC-MS) mixture of **4a-d** (344 mg, 1.2 mmol, 7% yield over two steps, 96.0% mixture purity + 3% overchlorinated byproducts) as an off-white solid (sample 3).

(3*R*,4*R*,7*S*,8*S*)-3,4,7,8-Tetrachlorodecane (**4a**): M.p. 102.1–102.8 °C. ^1^H NMR (600 MHz, CDCl_3_) *δ* 4.14–4.05 (m, 2H), 3.99 (app. dd, *J* = 9.5, 4.2, 1H), 3.98 (app. dd, *J* = 9.5, 4.2, 1H), 2.32–2.20 (m, 2H), 2.04–1.96 (m, 2H), 1.93–1.87 (m, 2H), 1.87–1.80 (m, 2H) 1.08 (t, *J* = 7.3 Hz, 6H). ^13^C NMR (151 MHz, CDCl_3_) *δ* 67.0, 64.8, 32.3, 27.6, 11.2. IR (ATR) (cm^−1^): 2960, 2934, 2873, 1464, 1432, 1382, 1294, 1282, 1208, 1110, 929, 878, 735, 676, 652, 621. Anal. Calcd. For C_10_H_18_Cl_4_: C, 42.89; H 6.48; Cl, 50.63. Found: C, 42.95; H, 6.50; Cl, 50.55. GC-MS: 99.3%, *t*_R_ = 16.14 min.

(3R*,4R*,7R*,8R*)-3,4,7,8-Tetrachlorodecane (**4b**) (extracted signals from sample 2): ^1^H NMR (600 MHz, CDCl_3_) *δ* 4.14–4.06 (m, 2H), 4.02–3.95 (m, 2H), 2.14–1.97 (m, 4H), 1.88–1.77 (m, 2H), 1.08 (t, *J* = 7.3 Hz, 6H). ^13^C NMR (151 MHz, CDCl_3_) *δ* 67.2, 64.1, 31.5, 27.7, 11.4.

## Author contribution statement

Solveig Valderhaug, Jiří Tůma: Conceived and designed the experiments; Performed the experiments; Analyzed and interpreted the data; Contributed reagents, materials, analysis tools or data; Wrote the paper.

Natalie Paškanová, Jana Herciková, Václav Eigner: Conceived and designed the experiments; Performed the experiments; Analyzed and interpreted the data; Contributed reagents, materials, analysis tools or data.

Huiling Liu, Jon Eigill Johansen, Odd Reidar Gautun: Conceived and designed the experiments; Wrote the paper.

Alexey Gorovoy: Conceived and designed the experiments; Performed the experiments.

## Data availability statement

Data is included in the article and supporting information. Supporting information contains NMR spectra and GC-MS data.

## Declaration of competing interest

The authors declare that they have no known competing financial interests or personal relationships that could have appeared to influence the work reported in this paper.

## References

[1] El-Shahawi M.S., Hamza A., Bashammakh A.S., Al-Saggaf W.T. (2010). An overview on the accumulation, distribution, transformations, toxicity and analytical methods for the monitoring of persistent organic pollutants. Talanta.

[2] Gobas F.A., de Wolf W., Burkhard L.P., Verbruggen E., Plotzke K. (2009). Revisiting bioaccumulation criteria for POPs and PBT assessments, integr. Environ. Assess. Manag..

[3] United Nations Treaty Collection (2001). https://treaties.un.org/Pages/ViewDetails.aspx?src=IND&mtdsg_no=XXVII-15&chapter=27&clang=_en.

[4] (2019). Stockholm Convention on Persistent Organic Pollutants (POPs).

[5] Vetter W., Sprengel J., Krätschmer K. (2022). Chlorinated paraffins – a historical consideration including remarks on their complexity. Chemosphere.

[6] Tomy G.T., Boer J. (2010). Chlorinated Paraffins.

[7] Kalinowska K., Lenartowicz P., Namieśnik J., Marć M. (2019). Analytical procedures for short chain chlorinated paraffins determination - how to make them greener?. Sci. Total Environ..

[8] van Mourik L.M., van der Veen I., Crum S., de Boer J. (2018). Developments and interlaboratory study of the analysis of short-chain chlorinated paraffins. Trends Anal. Chem..

[9] Mézière M., Cariou R., Larvor F., Bichon E., Guitton Y., Marchand P., Dervilly G., Le Bizec B. (2020). Optimized characterization of short-, medium, and long-chain chlorinated paraffins in liquid chromatography-high resolution mass spectrometry. J. Chom. A.

[10] van Mourik L.M., Lava R., O'Brien J., Leonards P.E.G., de Boer J., Ricci M. (2020). The underlying challenges that arise when analysing short-chain chlorinated paraffins in environmental matrices. J. Chromatogr., A.

[11] Schinkel L., Bogdal C., Canonica E., Cariou R., Bleiner D., McNeill K., Heeb N.V. (2018). Analysis of medium-chain and long-chain chlorinated paraffins: the urgent need for more specific analytical standards. Environ. Sci. Technol. Lett..

[12] Fernandes A.R., Vetter W., Dirks C., van Mourik L., Cariou R., Sprengel J., Heeb N., Lentjes A., Krätschmer K. (2022). Determination of chlorinated paraffins (CPs): analytical conundrums and the pressing need for reliable and relevant standards. Chemosphere.

[13] Frenzen G., Sippel H., Coelhan M. (1999). The relative configuration of a stereoisomer of 1,2,5,6,9,10-hexachlorodecane. Acta Crystallogr. C.

[14] Coelhan M. (2003). Synthesis of several single C10, C11 and C12 chloroalkanes. Fresenius Environ. Bull..

[15] Knobloch M.C., Schinkel L., Kohler H.-P.E., Mathis F., Kern S., Bleiner D., Heeb N.V. (2021). Transformation of short-chain chlorinated paraffins and olefins with the bacterial dehalogenase LinB from Sphingobium Indicum – kinetic models for the homologue-specific conversion of reactive and persistent material. Chemosphere.

[16] Zhang Q., Wang J., Zhu J., Liu J., Zhang J., Zhao M. (2016). Assessment of the endocrine-disrupting effects of short-chain chlorinated paraffins in in vitro models. Environ. Int..

[17] Ali I., Gupta V.K., Aboul-Enein H.Y. (2003). Chirality: a challenge for the environmental scientists. Curr. Sci..

[18] Ali I., Aboul-Enein H.Y., Ghanem A. (2005). Enantioselective toxicity and carcinogenesis. Curr. Pharmaceut. Anal..

[19] West C. (2018). Current trends in supercritical fluid chromatography. Anal. Bioanal. Chem..

[20] Riddell N., van Bavel B., Jogsten I.E., McCrindle R., McAlees A., Chittim B. (2017). Coupling supercritical fluid chromatography to positive ion atmospheric pressure ionization mass spectrometry: ionization optimization of halogenated environmental contaminants. Int. J. Mass Spectrom..

[21] Hatzimarinaki M., Orfanopoulos M. (2006). Novel methodology for the preparation of five-, seven-, and nine-membered fused rings on C60. Org. Lett..

[22] Ishmuratov G.Y., Yakovleva M.P., Ganieva V.A., Kharisov R.Y., Gazetdinov R.R., Abulkaramova A.M., Tolstikov G.A. (2006). Synthesis of 3*S*-methylundec-1-ylbromide, a key synthon in the synthesis of (*S,S,S*)-diprionylacetate, from L-(-)-menthol. Chem. Nat. Compd..

[23] Farfán P., Gómez S., Restrepo A. (2019). Dissection of the mechanism of the Wittig reaction. J. Org. Chem..

[24] Bosshardt H., Schlosser M. (1980). Die Strukturdynamik von Pentadienylmetall-Verbindungen mit endständiger Alkyl-Gruppe: zugleich «stereoselektive» und «stereodefensive» Synthese eines natürlichen Riechstoffes. Helv. Chim. Acta.

[25] Patel N.R., Kelly C.B., Jouffroy M., Molander G.A. (2016). Engaging alkenyl halides with alkylsilicates via photoredox dual catalysis. Org. Lett..

[26] Burg F., Rovis T.J. (2021). Diastereoselective three-component 3,4-amino oxygenation of 1,3-dienes catalyzed by a cationic heptamethylindenyl rhodium(III) complex. J. Am. Chem. Soc..

[27] Roberts I., Kimball G.E. (1937). The halogenation of ethylenes. J. Am. Chem. Soc..

[28] Lerner L. (2011). Small-Scale Synthesis of Laboratory Reagents with Reaction Modeling.

[29] Pedersen D.S., Rosenbohm C. (2001). Dry column vacuum chromatography. Synthesis.

[30] Bruker (2021).

[31] Palatinus L., Chapuis G. (2007). SUPERFLIP–a computer program for the solution of crystal structures by charge flipping in arbitrary dimensions. J. Appl. Cryst.

[32] Betteridge P.W., Carruthers J.R., Cooper R.I., Prout K., Watkin D.J. (2003). CRYSTALS version 12: software for guided crystal structure analysis. J. Appl. Cryst.

[33] (1999). ^1^H NMR Spectrum; Triphenylpropylphosphonium Bromide.

[34] Moret E., Desponds O., Schlosser M. (1991). 1,(ω - 1)-Dienes: solvent controlled unilateral or bilateral metalation. J. Organomet. Chem..

