# Supplementary material for: Synthesis, identification, chiral separation and crystal structure of (3R,4R,7S,8S)-3,4,7,8-tetrachlorodecane and its stereoisomers
Source: Heliyon. 2023 Jun 3;9(6):e16987. doi: 10.1016/j.heliyon.2023.e16987 (PMC10279909; doi:10.1016/j.heliyon.2023.e16987)
Supplement: Multimedia component 1 [file mmc1.docx]

***Supporting Information***

**Synthesis, identification, chiral separation and crystal structure of (3*R*,4*R*,7*S*,8*S*)-3,4,7,8-tetrachlorodecane and its stereoisomers**

Solveig Valderhaug^a,b^, Natalie Paškanová^c^, Jiří Tůma^b,c^, Jana Herciková^c^, Václav Eigner^d^, Huiling Liu^b^, Alexey Gorovoy^b^, Jon Eigill Johansen^b^, Odd Reidar Gautun^a^*

^a^ Department of Chemistry, Norwegian University of Science and Technology (NTNU), Høgskoleringen 5, NO-7491, Trondheim, Norway

^b^ Chiron AS, Stiklestadveien 1, NO-7041 Trondheim, Norway

^c^ Department of Organic Chemistry, University of Chemistry and Technology, Technická 5, 16628, Prague, Czech Republic

^d^ Department of Solid State Chemistry, University of Chemistry and Technology, Technická 5, 16628, Prague, Czech Republic

1 NMR and GC-MS data 2

1.1 Propyltriphenylphosphonium bromide (**2**) 2

1.2 Deca-3,7-diene (**3**) 2

1.3 (3*R*,4*R*,7*S*,8*S*)-Tetrachlorodecane (**4a**) – sample 1 3

1.4 3,4,7,8-Tetrachlorodecane (**4**) – sample 2 8

1.5 3,4,7,8-Tetrachlorodecane (**4**) – sample 3 11

# NMR and GC-MS data

## Propyltriphenylphosphonium bromide (2)


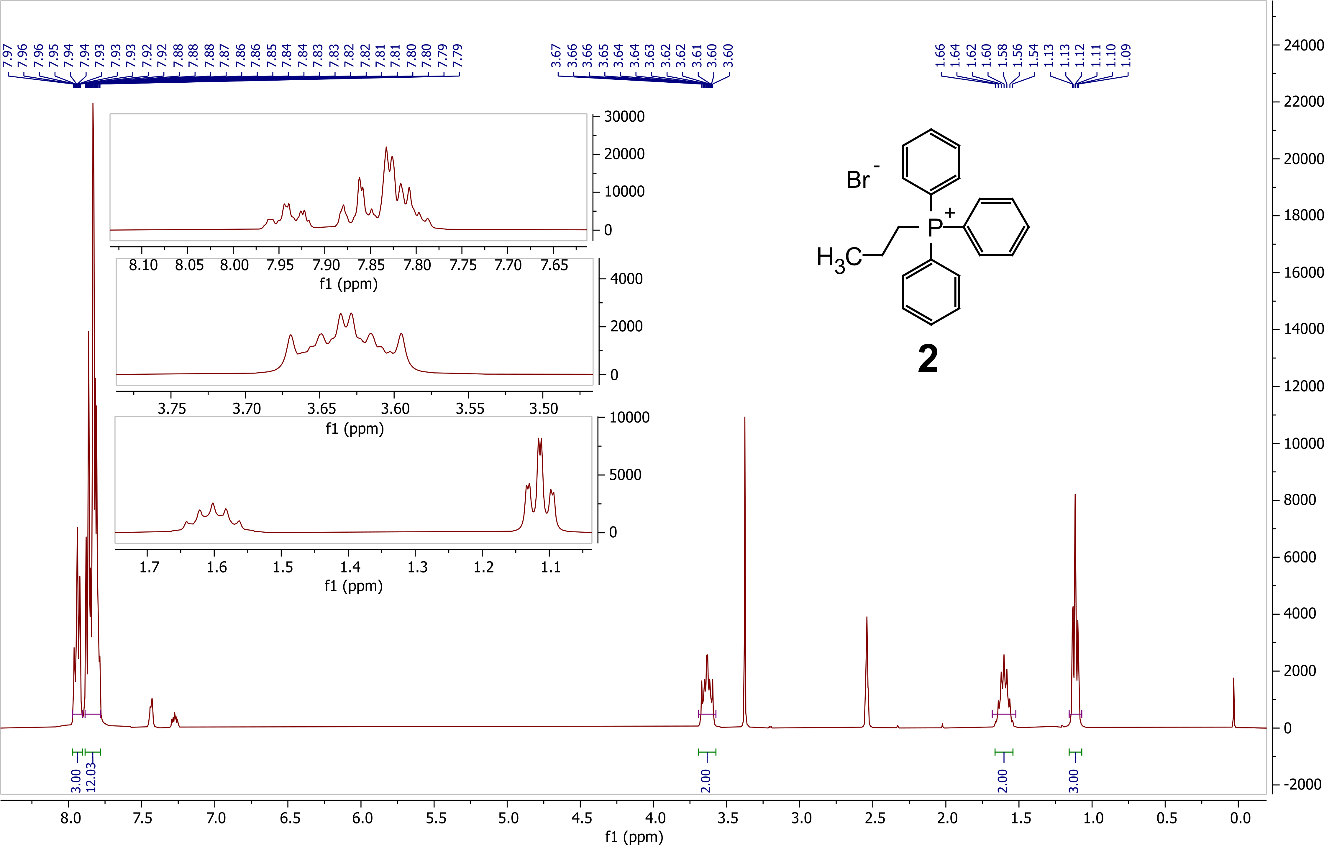


**Figure S1:** ^1^H NMR (400 MHz, DMSO-d_6_) of **2**.

## Deca-3,7-diene (3)


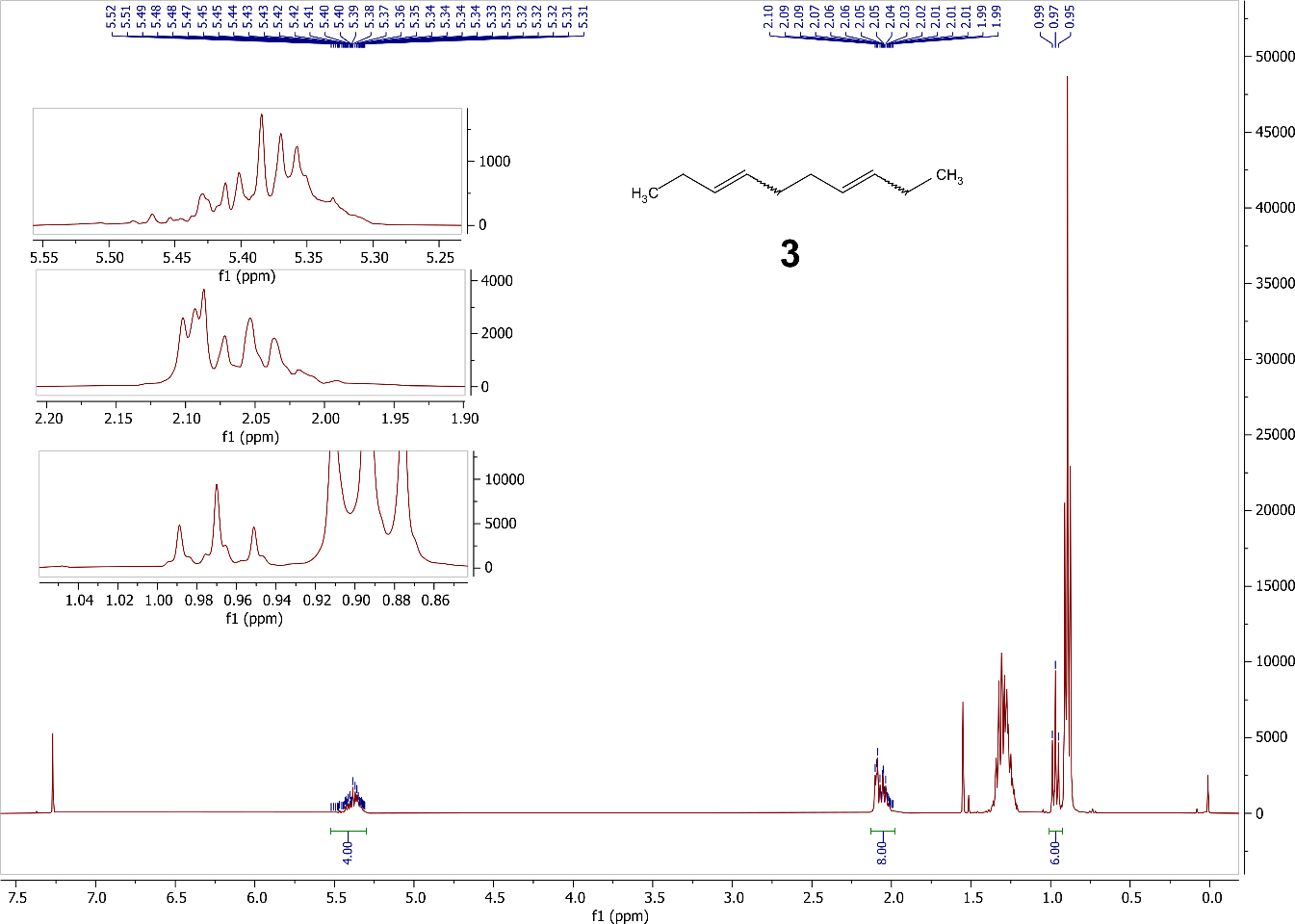


**Figure S2:** ^1^H NMR (400 MHz, CDCl_3_) of **3**.

## (3*R*,4*R*,7*S*,8*S*)-Tetrachlorodecane (4a) – sample 1


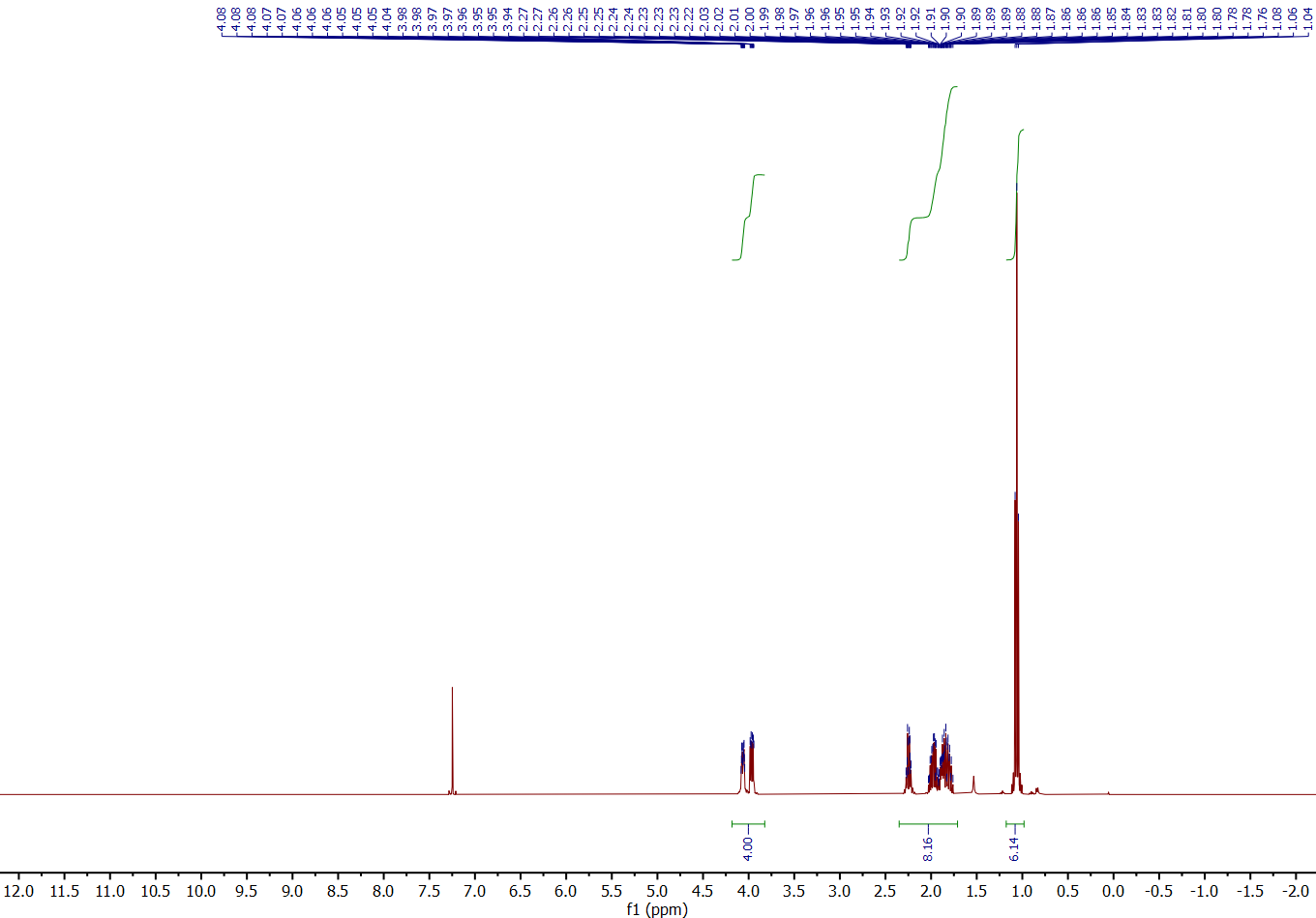


**Figure S3:** ^1^H NMR (400 MHz, CDCl_3_) of **4a**, sample 1, full spectral width.


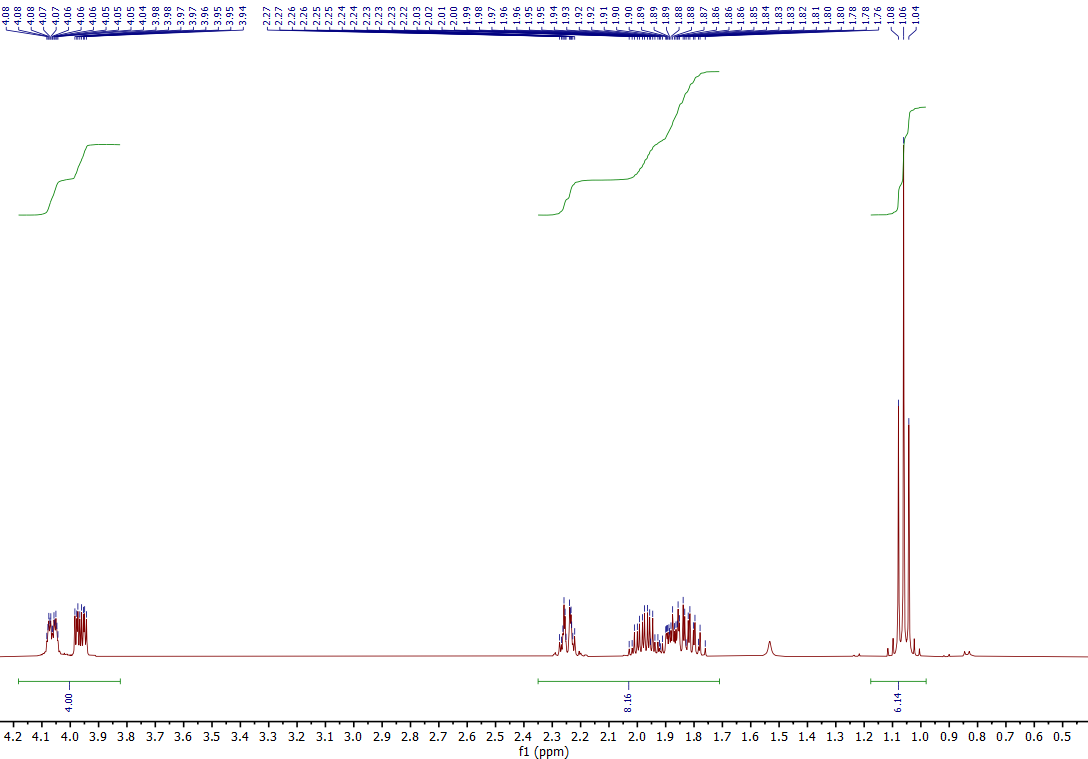


**Figure S4:** ^1^H NMR (400 MHz, CDCl_3_) of **4a**, sample 1, zoomed in.


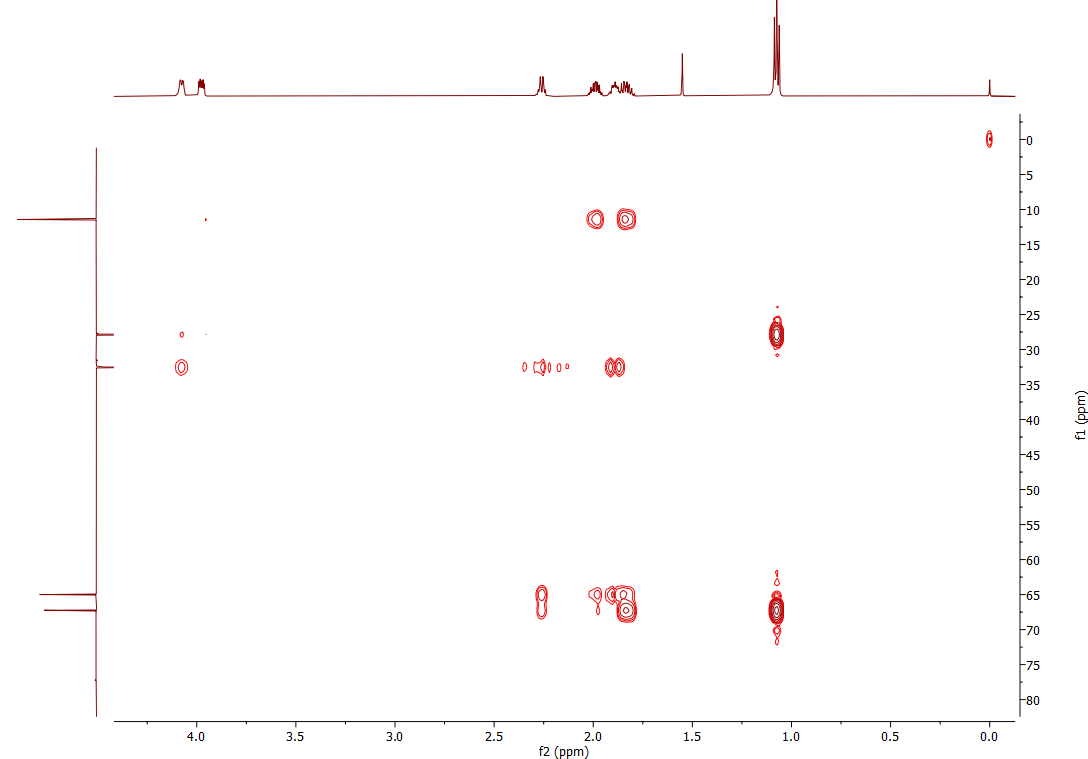


**Figure S5:** ^1^H-^1^H COSY NMR (600 MHz, CDCl_3_) of **4a**, sample 1, zoomed in.


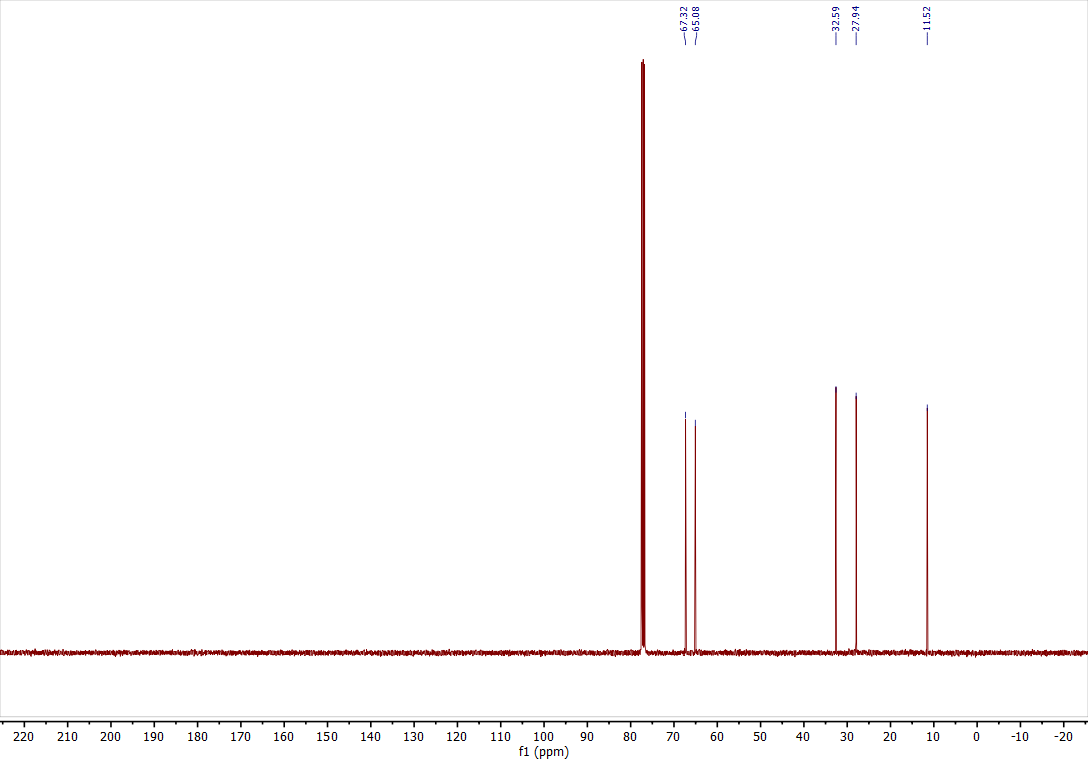


**Figure S6:** ^13^C (101 MHz, CDCl_3_) of **4a**, sample 1, full spectral width.


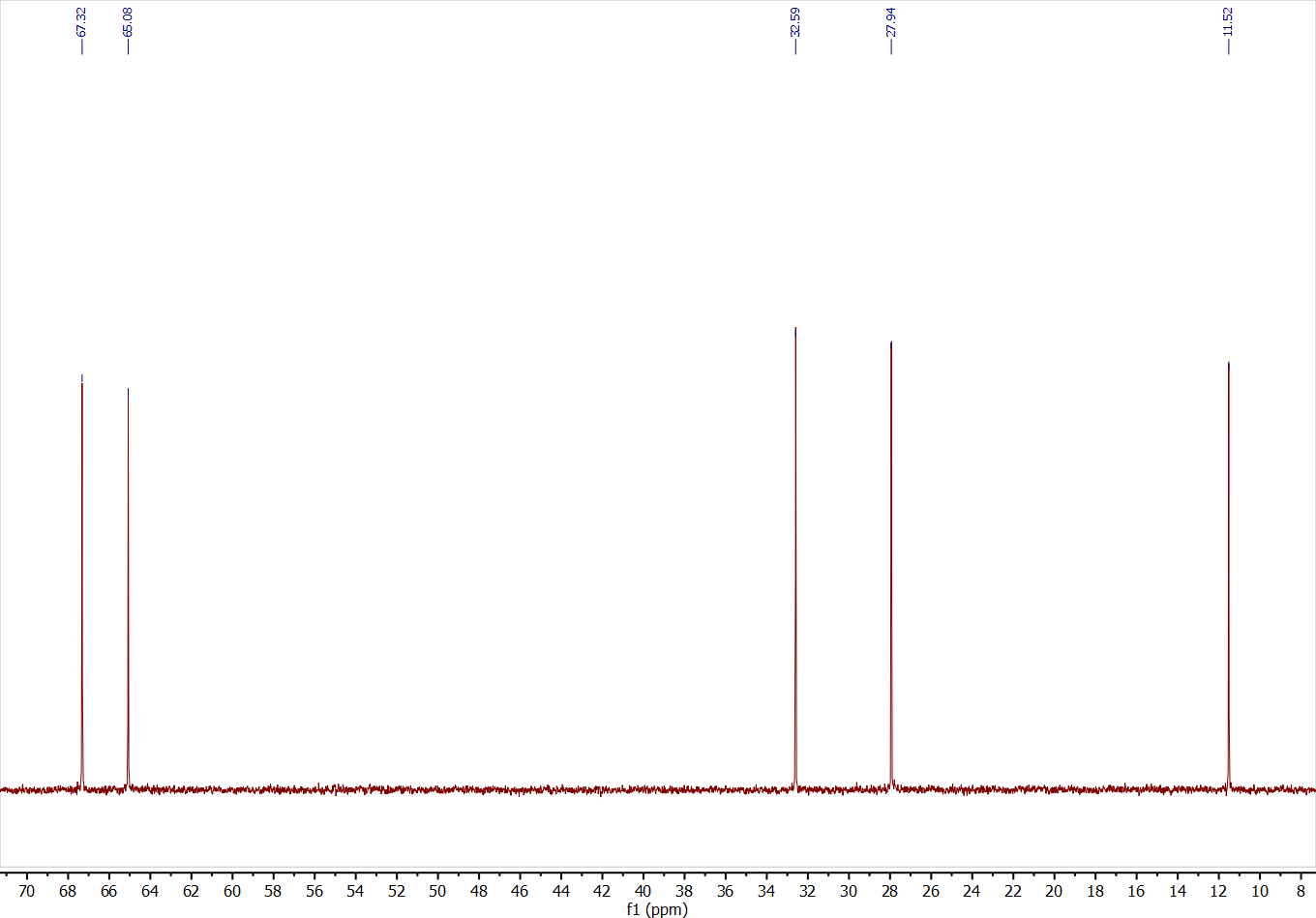


**Figure S7:** ^13^C (101 MHz, CDCl_3_) of **4a**, sample 1, zoomed in.


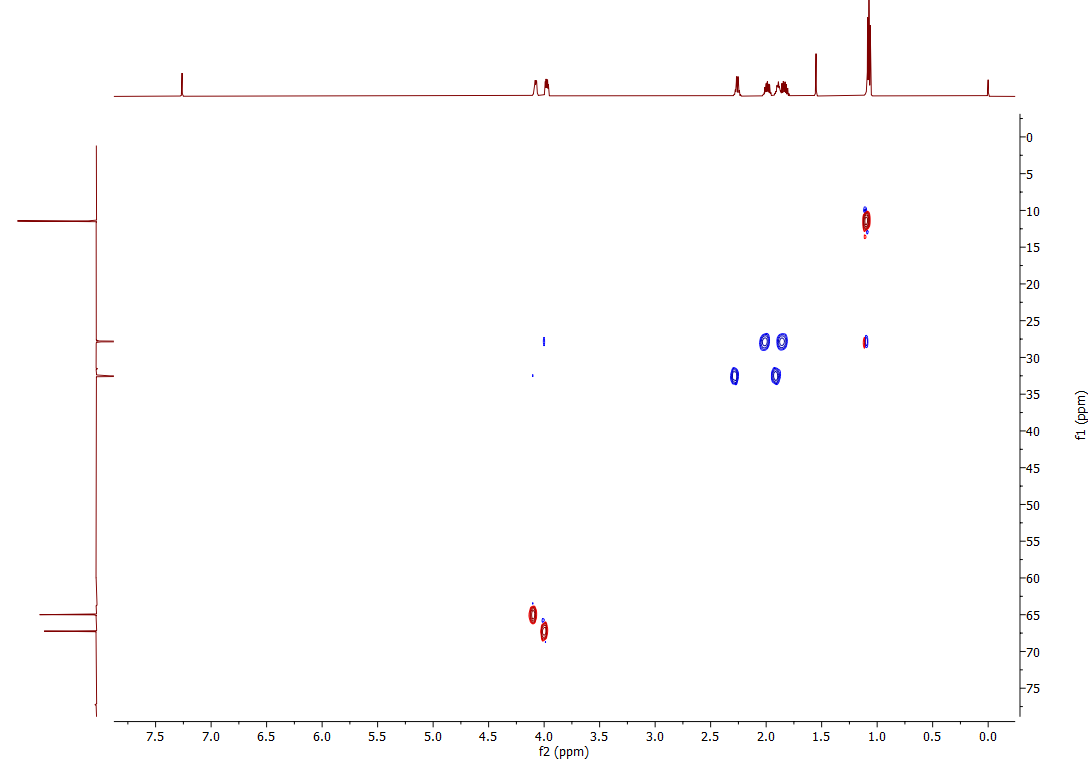


**Figure S8:** ^1^H -^13^C HSQC NMR (600/151 MHz, CDCl_3_) of **4a**, sample 1, zoomed in.


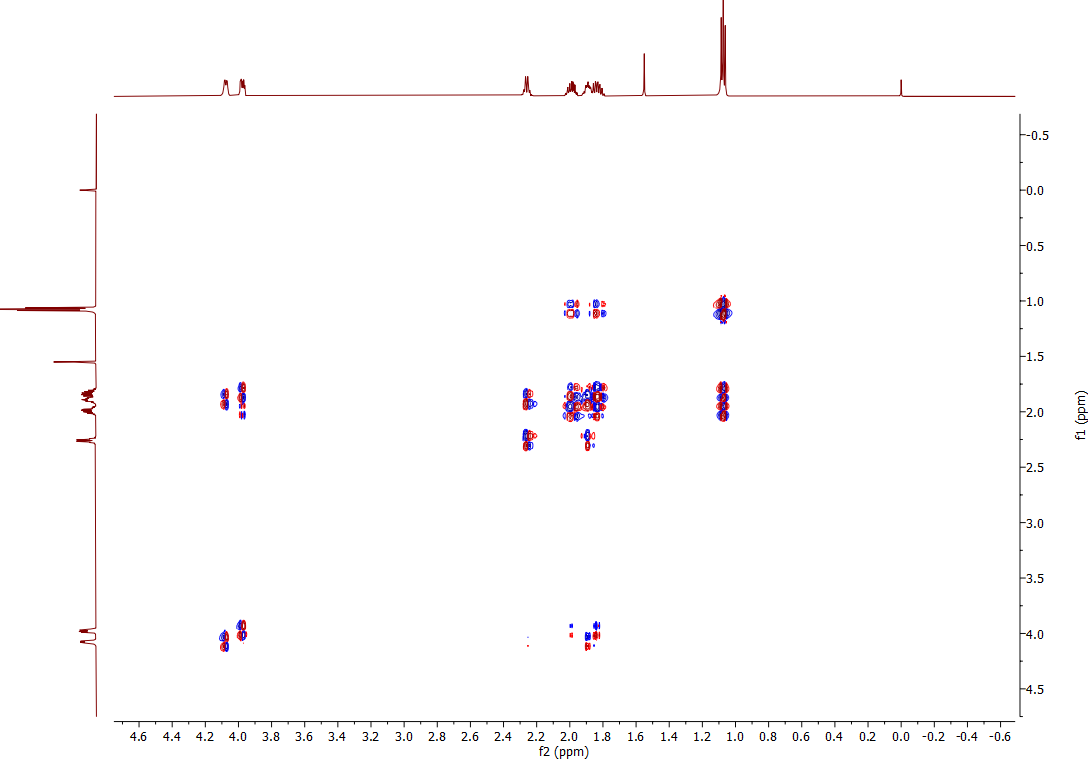


**Figure S9:** ^1^H -^13^C HMBC NMR (600/151 MHz, CDCl_3_) of **4a**, sample 1, zoomed in.


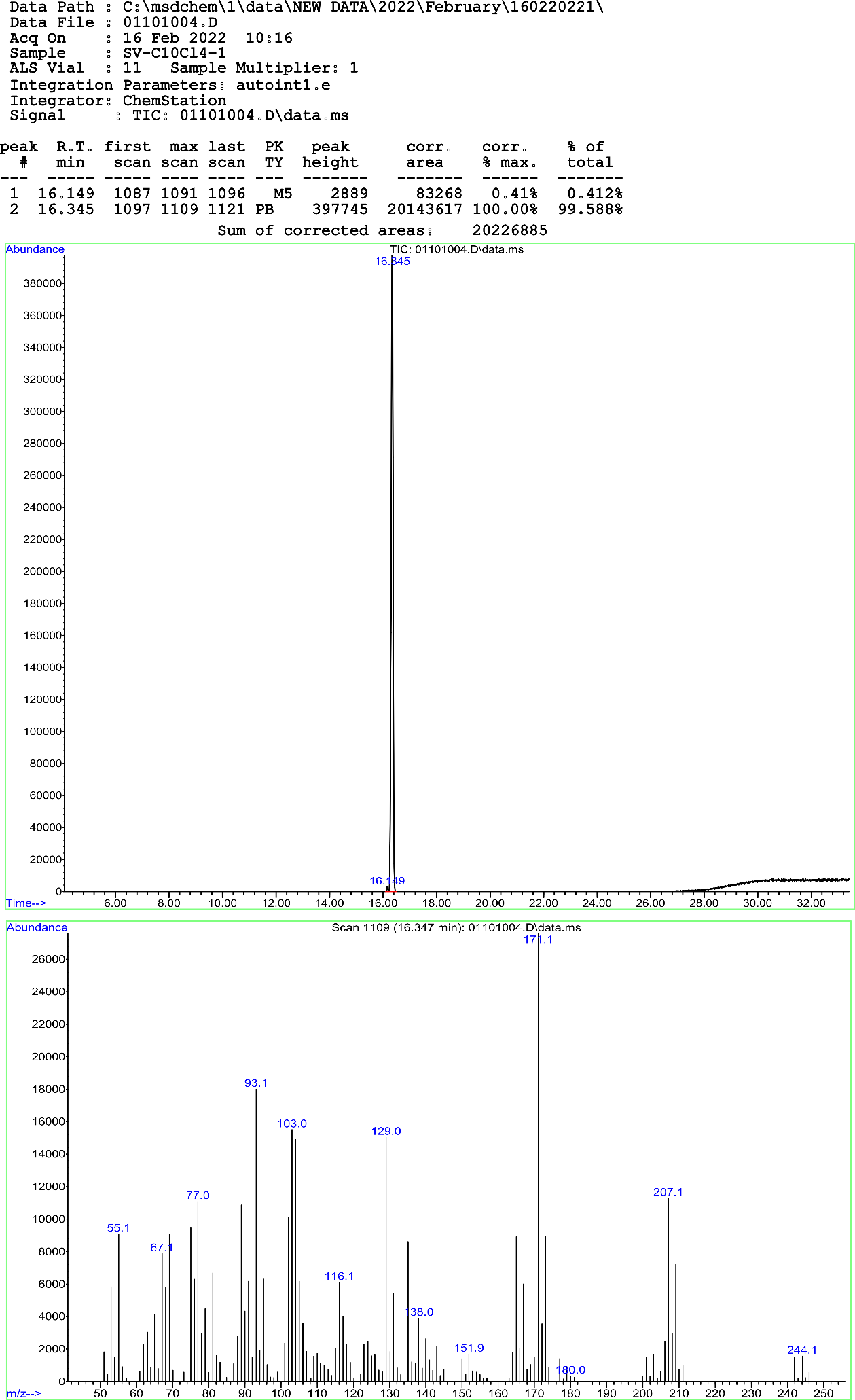


**Figure S10:** GC-MS of **4a**, sample 1.

## 3,4,7,8-Tetrachlorodecane (4) – sample 2


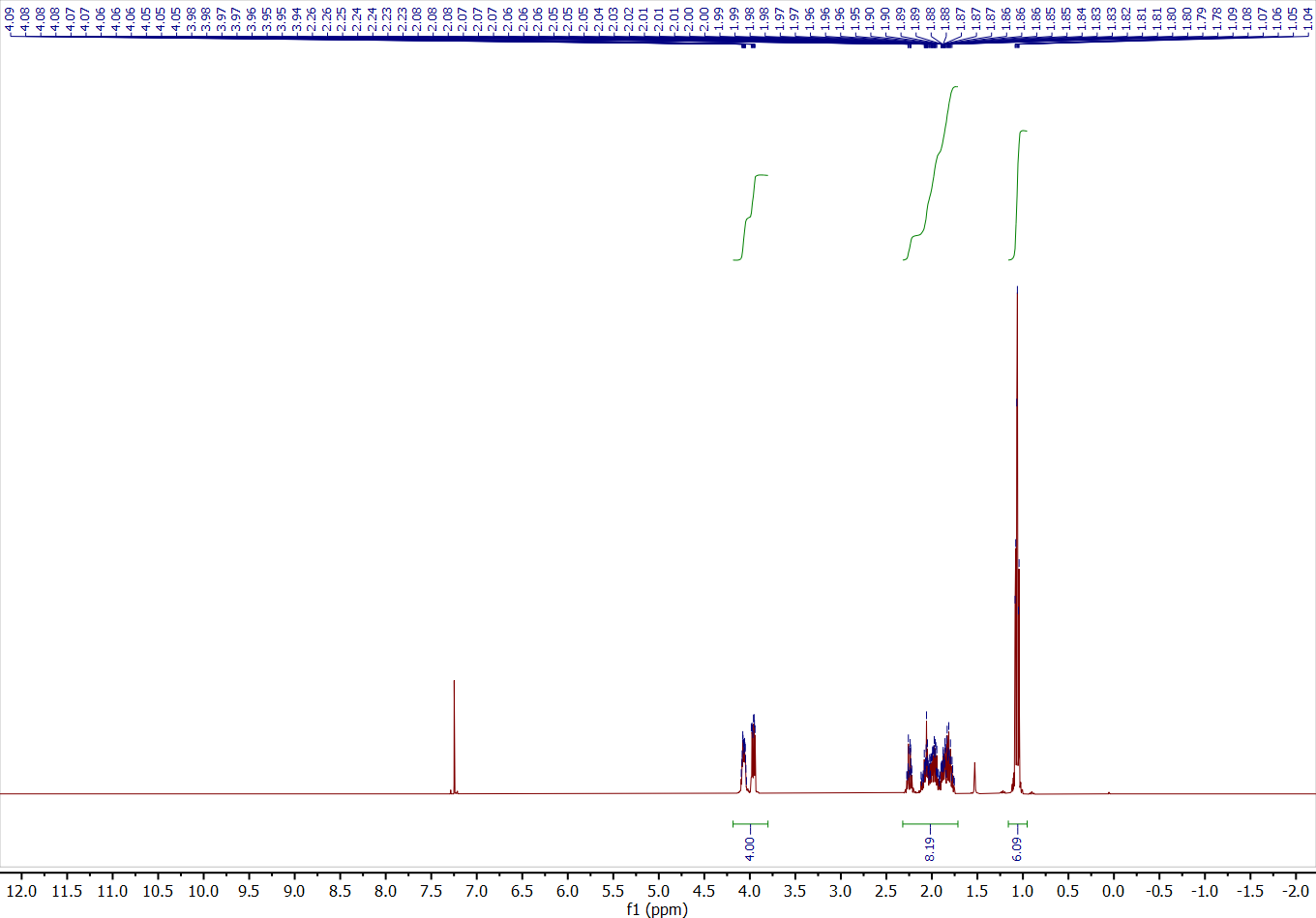


**Figure S11:** ^1^H NMR (400 MHz, CDCl_3_) of sample 2, full spectral width.


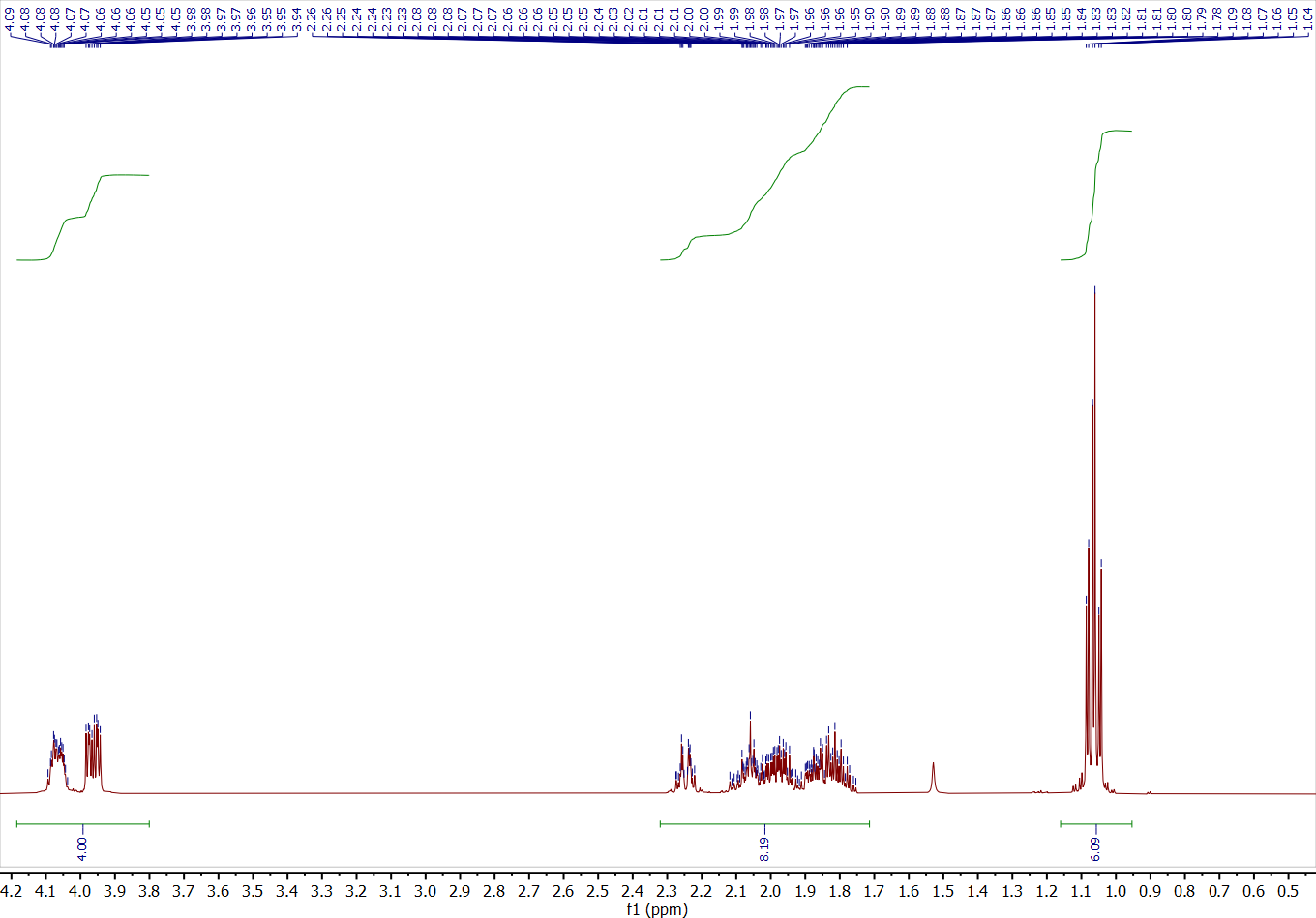


**Figure S12:** ^1^H NMR (400 MHz, CDCl_3_) of sample 2, zoomed in.


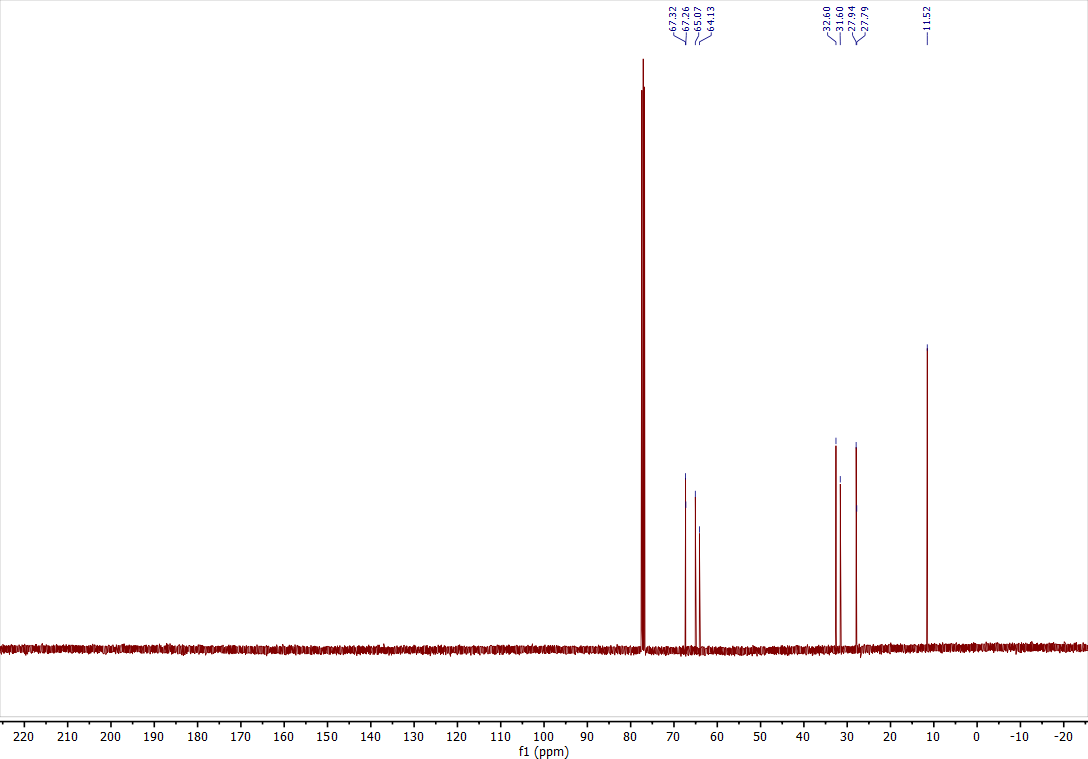


**Figure S13:** ^13^C (101 MHz, CDCl_3_) of sample 2, full spectral width.


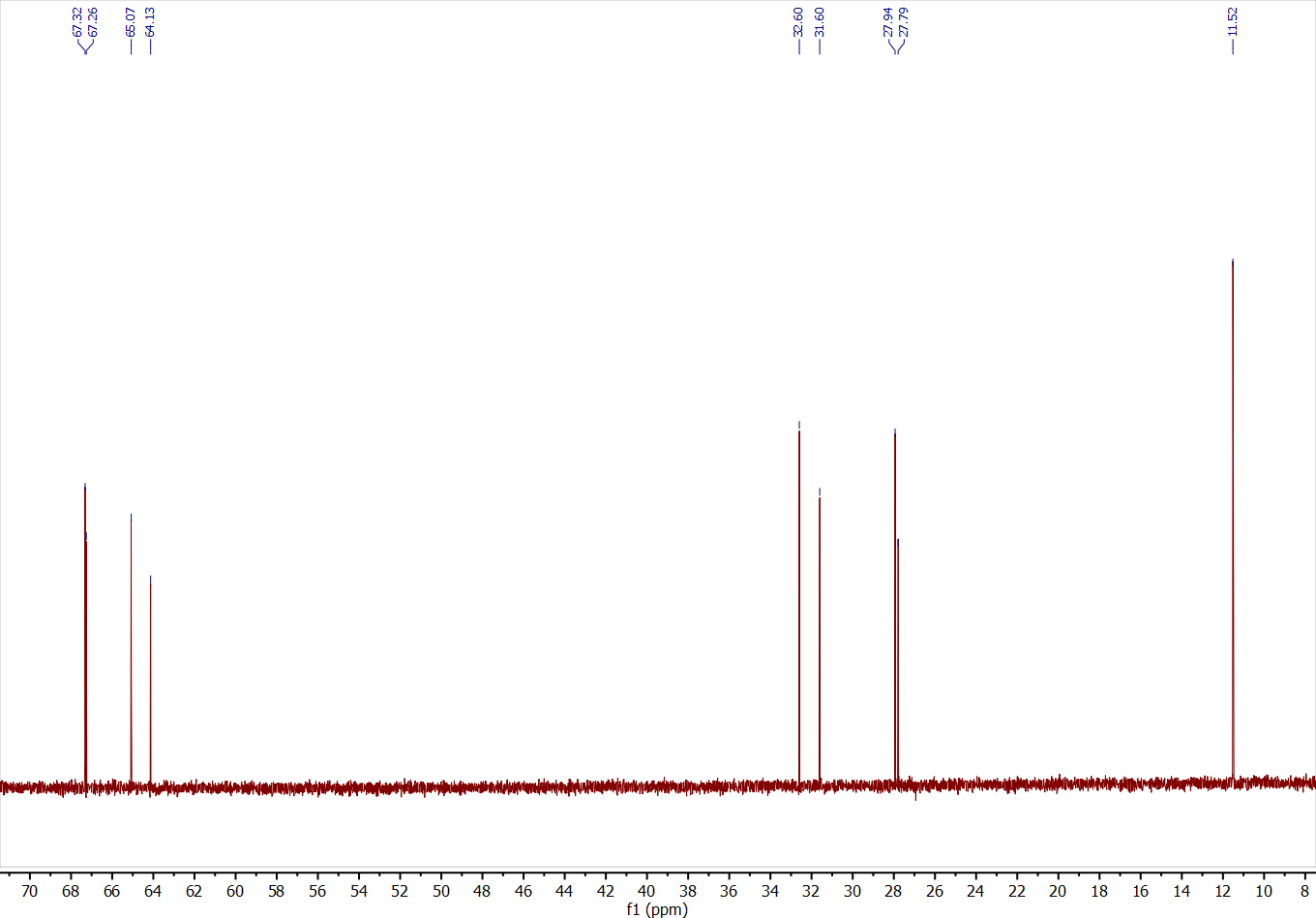


**Figure S14:** ^13^C (101 MHz, CDCl_3_) of sample 2, zoomed in.


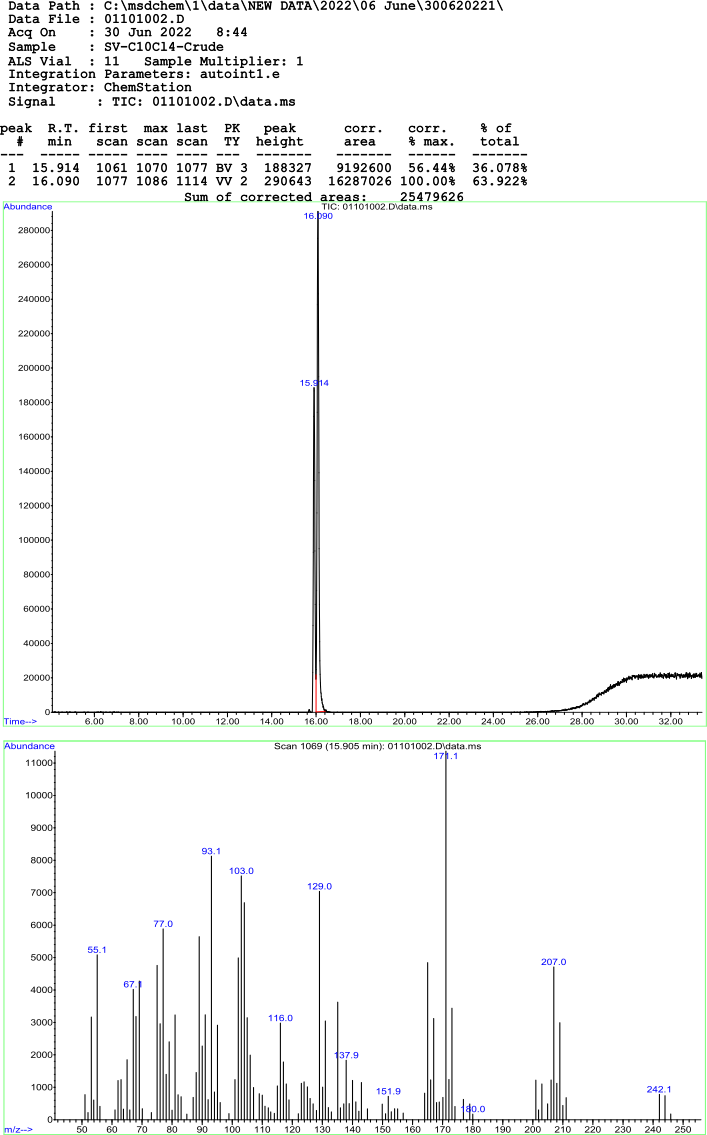


**Figure S15:** GC-MS of sample 2.

## 3,4,7,8-Tetrachlorodecane (4) – sample 3


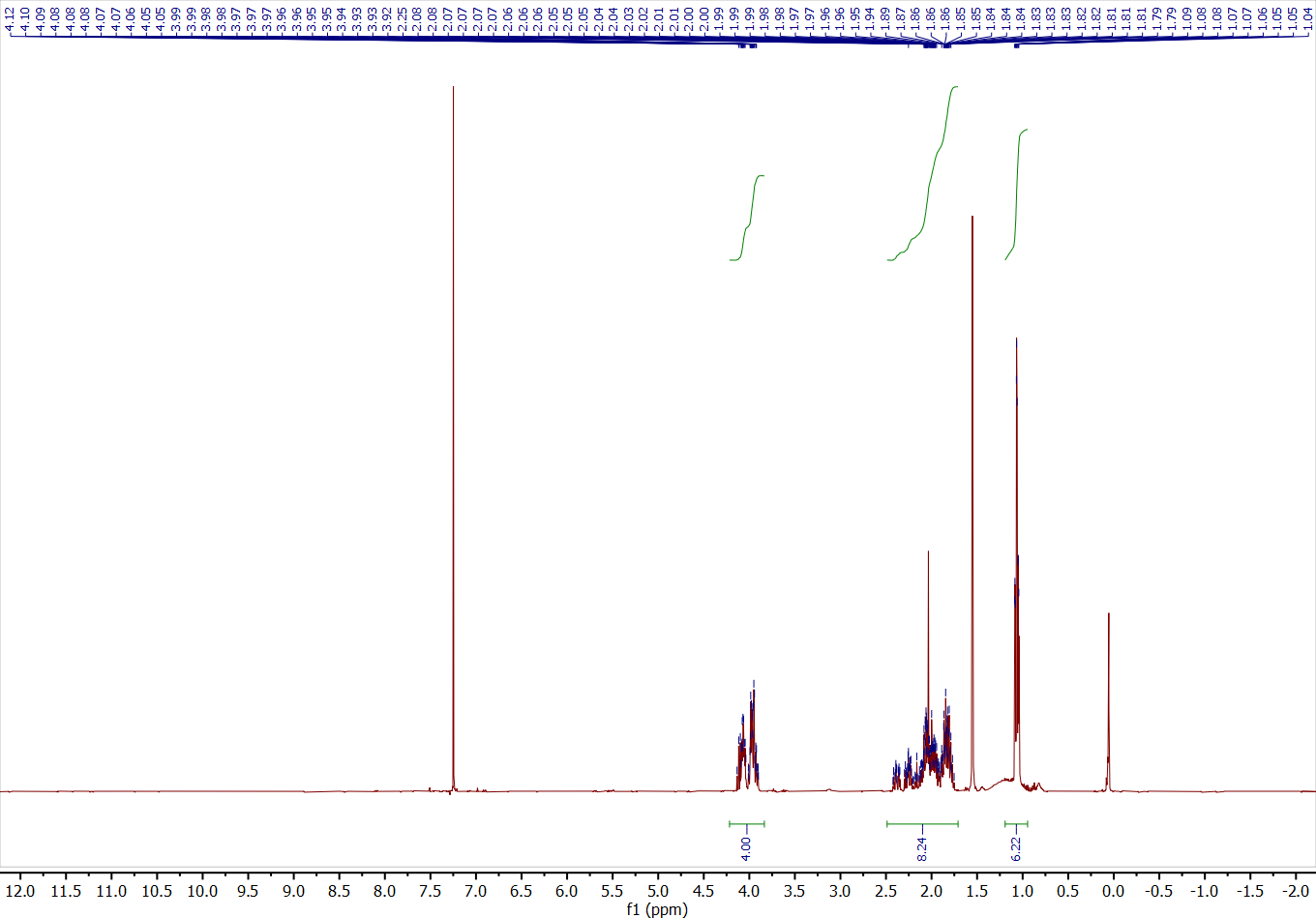


**Figure S16:** ^1^H NMR (400 MHz, CDCl_3_) of sample 3, full spectral width.


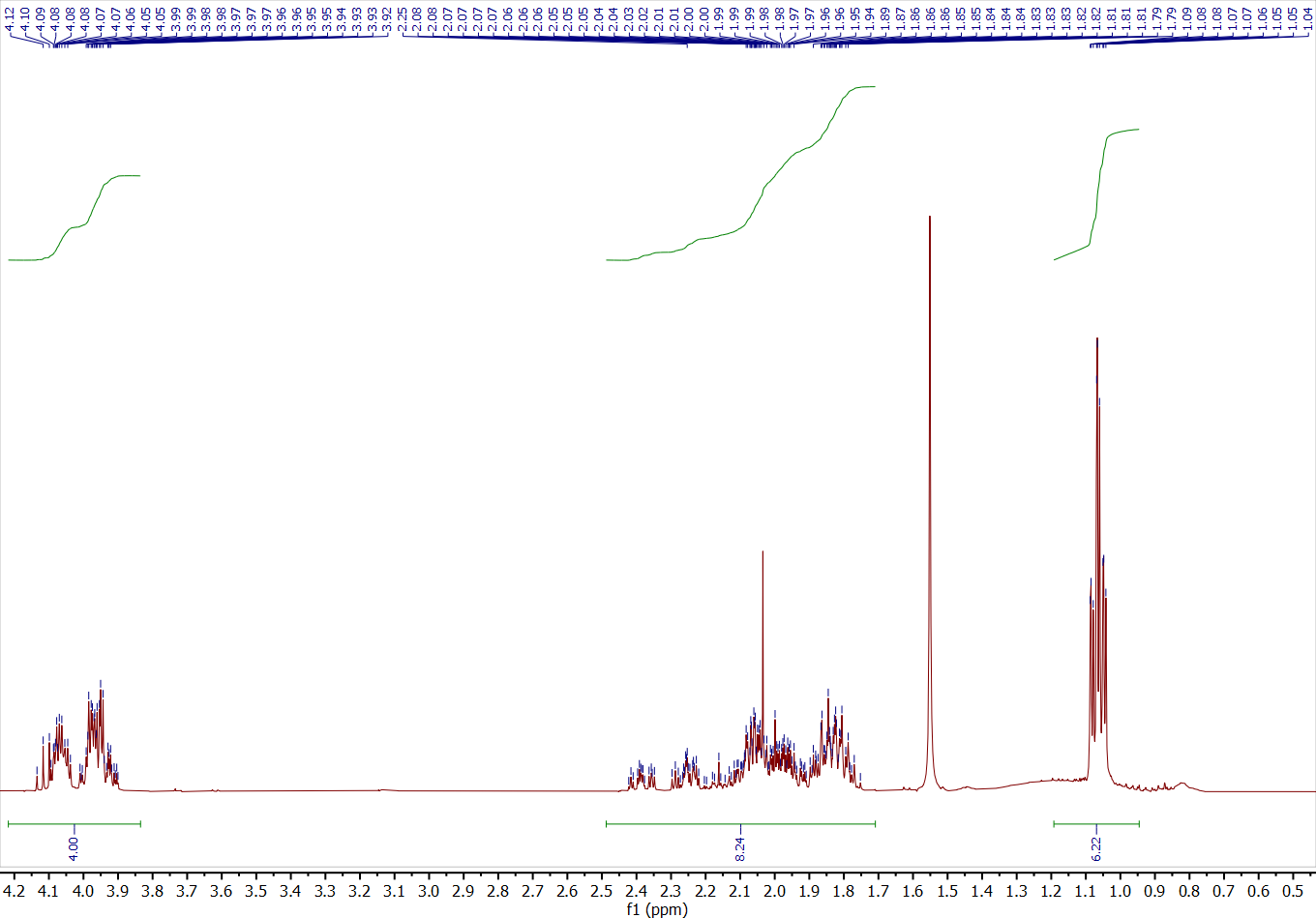


**Figure S17:** ^1^H NMR (400 MHz, CDCl_3_) of sample 3, zoomed in.


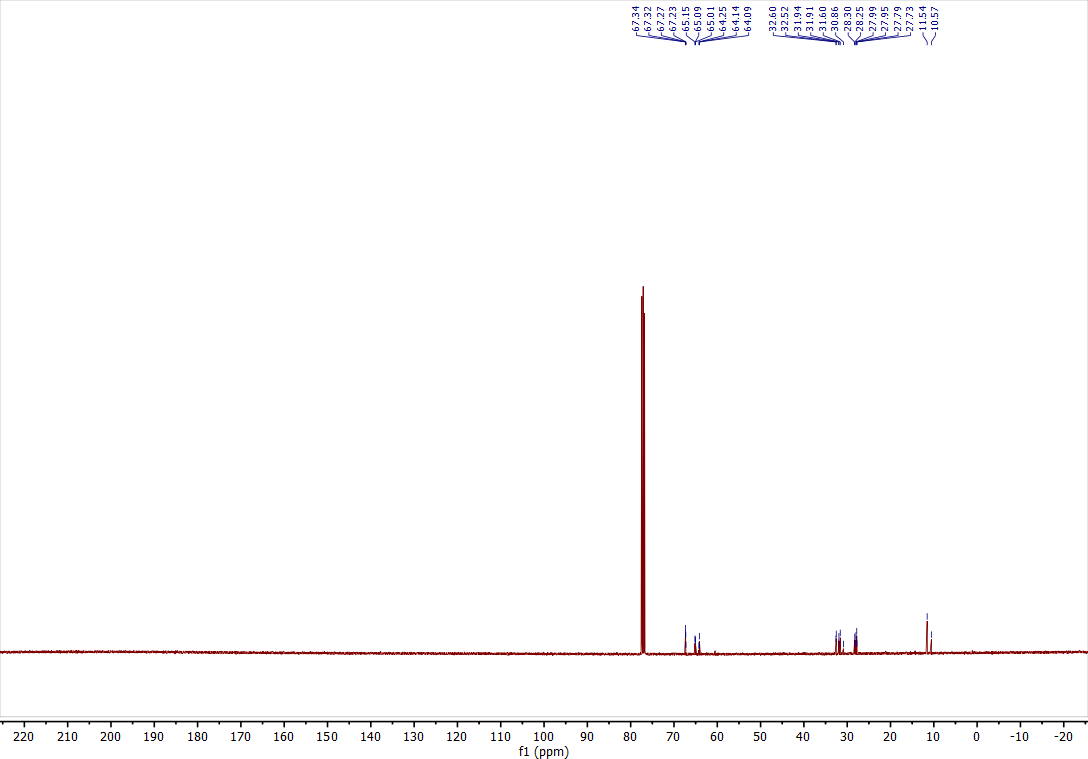


**Figure S18:** ^13^C NMR (101 MHz, CDCl_3_) of sample 3, full spectral width.


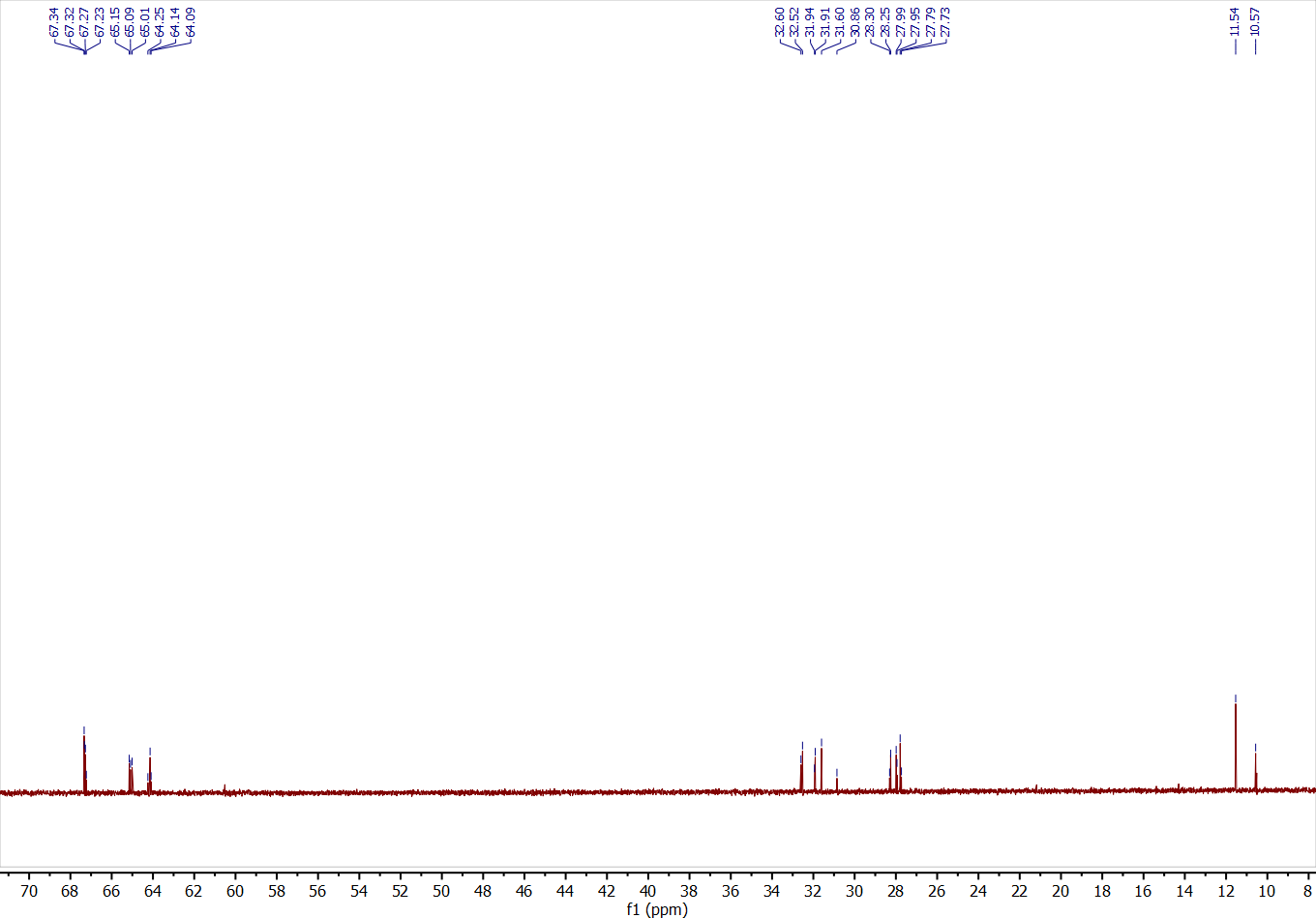


**Figure S19:** ^13^C NMR (101 MHz, CDCl_3_) of sample 3, zoomed in.


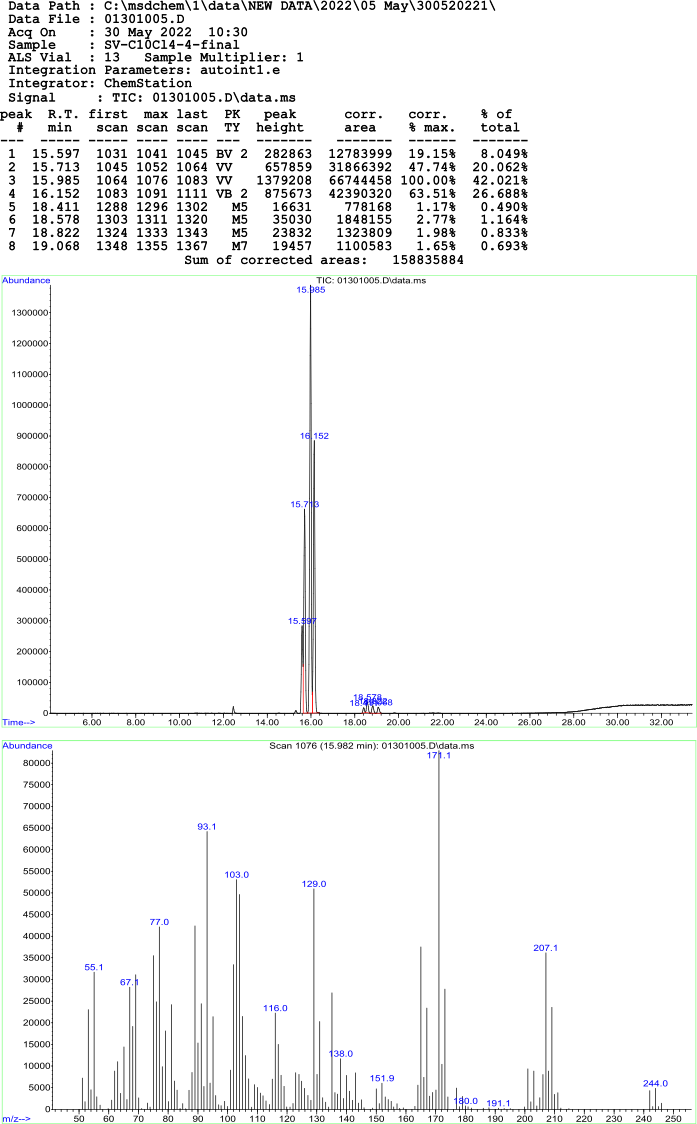


**Figure S20:** GC-MS of sample 3.
